# Supplementary material for: Phylobone: a comprehensive database of bone extracellular matrix proteins in human and model organisms
Source: Bone Res. 2023 Aug 15;11:44. doi: 10.1038/s41413-023-00281-w (PMC10425349; doi:10.1038/s41413-023-00281-w)
Supplement: Supplementary file 1 — Supplementary Material [file 41413_2023_281_MOESM1_ESM.pdf]

SUPPLEMENTARY MATERIAL

Phylobone: a comprehensive database of bone extracellular matrix (ECM) proteins in human and model organisms

Margalida Fontcuberta-Rigo[1], Miho Nakamura[1,2,3,*] and Pere Puigbò[4,5,6,*]

[1] Medicity Research Laboratory, Faculty of Medicine, University of Turku, Tykistökatu 6, 20520 Turku, Finland.
[2] Institute of Biomaterials and Bioengineering, Tokyo Medical and Dental University, 2-3-10 Kanda-Surugadai, Chiyoda, Tokyo 1010062 Japan
[3] Graduate School of Engineering, Tohoku University, 6-6 Aramaki Aza Aoba, Aoba-ku, Sendai, Miyagi 9808579 Japan
[4] Department of Biology, University of Turku, 20500 Turku, Finland
[5] Nutrition and Health Unit, Eurecat Technology Centre of Catalonia, 43204 Reus, Catalonia, Spain.
[6] Department of Biochemistry and Biotechnology, Rovira i Virgili University, 43007 Tarragona, Catalonia, Spain

* To whom correspondence should be addressed. Email: pepuav@utu.fi (P.P.), miho.nakamura@utu.fi (M.N.)

SUPPLEMENTARY FIGURES

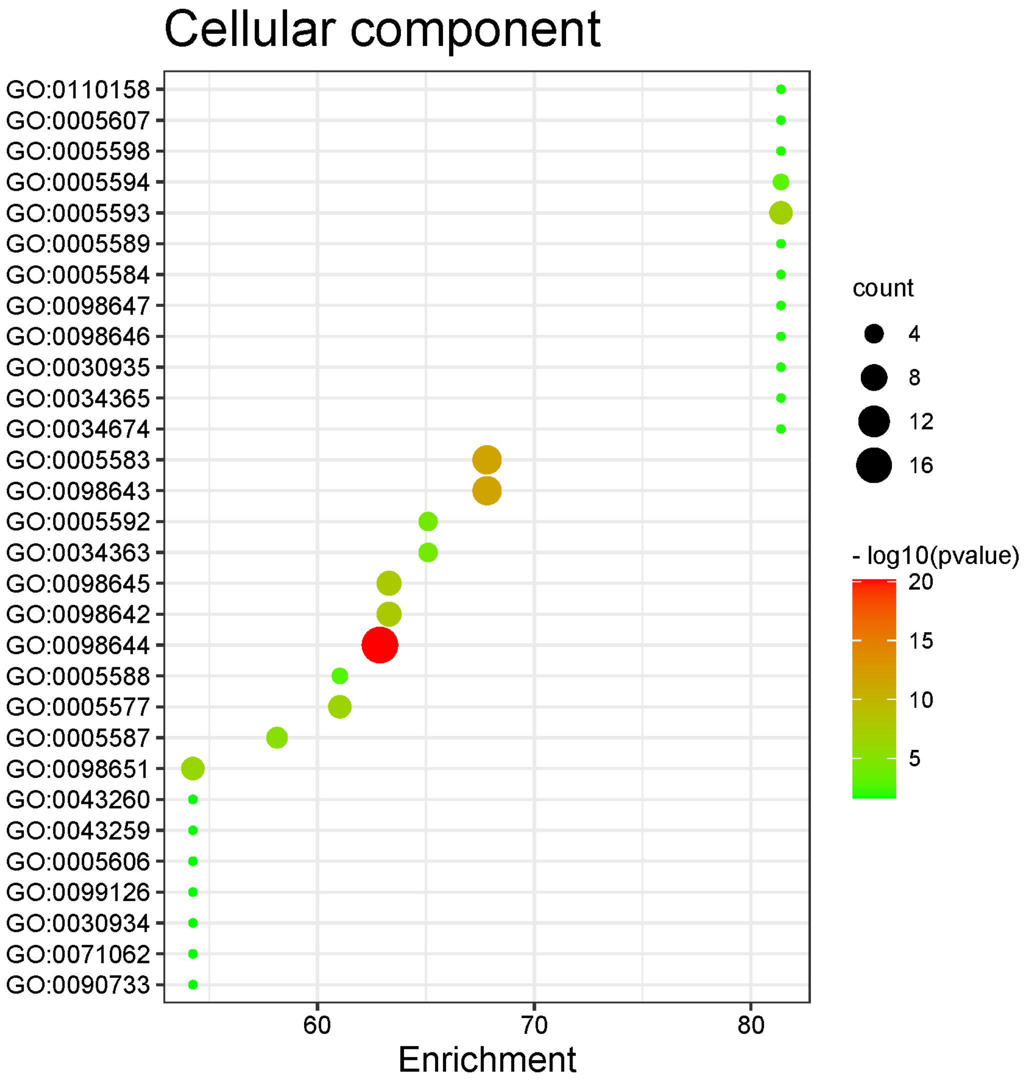

Supplementary figure S1. Representation of cellular components GO categories with higher enrichment in bone ECM proteins of *Homo sapiens*.
GO:0110158, calpain complex; GO:0005607, laminin-2 complex; GO:0005598, short-chain collagen trimer; GO:0005594, collagen type IX trimer; GO:0005593, FACIT collagen trimer; GO:0005589, collagen type VI trimer; GO:0005584, collagen type I trimer; GO:0098647, collagen beaded filament; GO:0098646, collagen sheet; GO:0030935, sheet-forming collagen trimer; GO:0034365, discoidal high-density lipoprotein particle; GO:0034674, integrin alpha5-beta1 complex; GO:0005583, fibrillar collagen trimer; GO:0098643, banded collagen fibril; GO:0005592, collagen type XI trimer; GO:0034363, intermediate-density lipoprotein particle; GO:0098645, collagen network; GO:0098642, network-forming collagen trimer; GO:0098644, complex of collagen trimers; GO:0005588, collagen type V trimer; GO:0005577, fibrinogen complex; GO:0005587, collagen type IV trimer; GO:0098651, basement membrane collagen trimer; GO:0043260, laminin-11 complex; GO:0043259, laminin-10 complex; GO:0005606, laminin-1 complex; GO:0099126, transforming growth factor beta complex; GO:0030934, anchoring collagen complex; GO:0071062, alphav-beta3 integrin-vitronectin complex; and GO:0090733, tenascin complex.

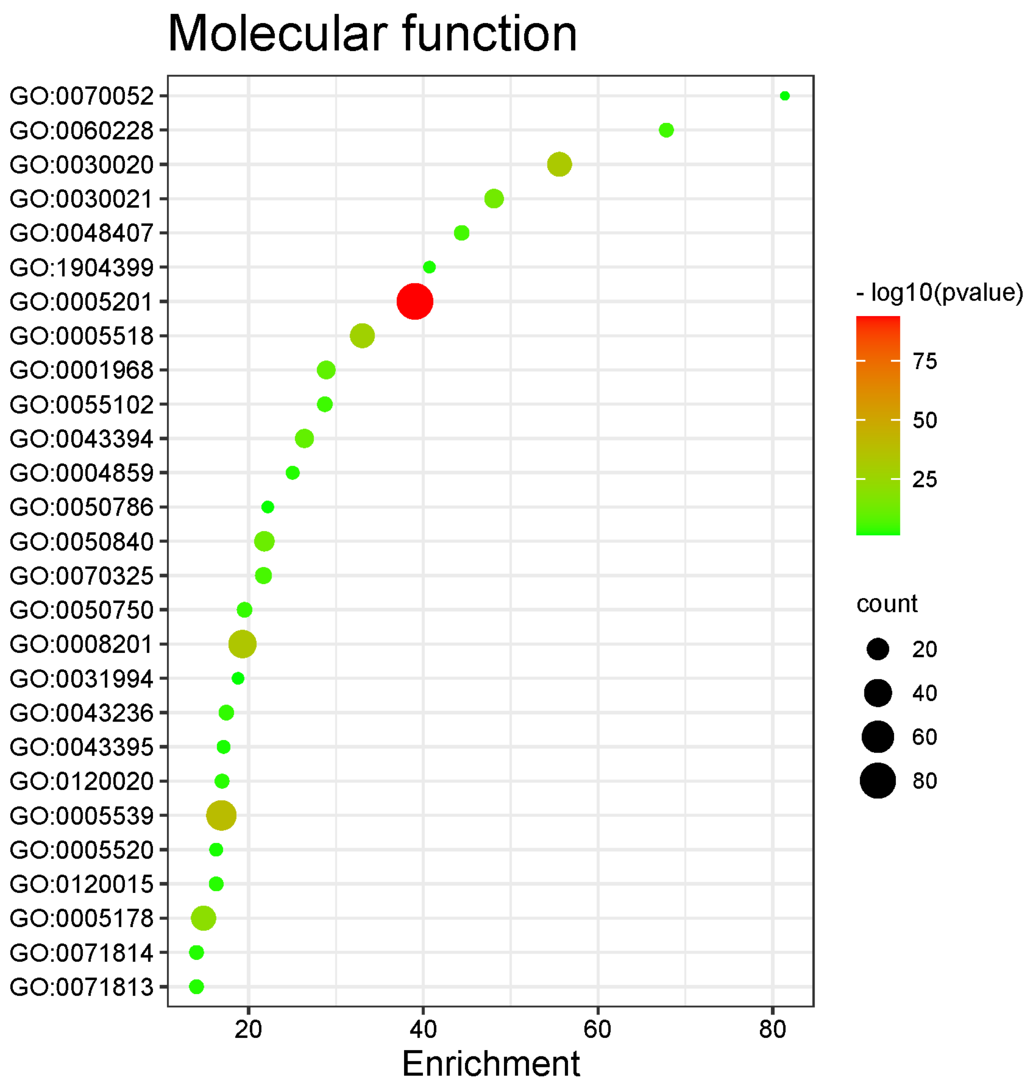

Supplementary figure S2. Representation of molecular function GO categories with higher enrichment in bone ECM proteins of *Homo sapiens*.
GO:0070052, collagen V binding; GO:0060228, phosphatidylcholine-sterol O-acyltransferase activator activity; GO:0030020, extracellular matrix structural constituent conferring tensile strength; GO:0030021, extracellular matrix structural constituent conferring compression resistance; GO:0048407, platelet-derived growth factor binding; GO:1904399, heparan sulfate binding; GO:0005201, extracellular matrix structural constituent; GO:0005518, collagen binding; GO:0001968, fibronectin binding; GO:0055102, lipase inhibitor activity; GO:0043394, proteoglycan binding; GO:0004859, phospholipase inhibitor activity; GO:0050786, RAGE receptor binding; GO:0050840, extracellular matrix binding; GO:0070325, lipoprotein particle receptor binding; GO:0050750, low-density lipoprotein particle receptor binding; GO:0008201, heparin binding; GO:0031994, insulin-like growth factor I binding; GO:0043236, laminin binding; GO:0043395, heparan sulfate proteoglycan binding; GO:0120020, cholesterol transfer activity; GO:0005539, glycosaminoglycan binding; GO:0005520, insulin-like growth factor binding; GO:0120015, sterol transfer activity; GO:0005178, integrin binding; GO:0071814, protein-lipid complex binding; and GO:0071813, lipoprotein particle binding.

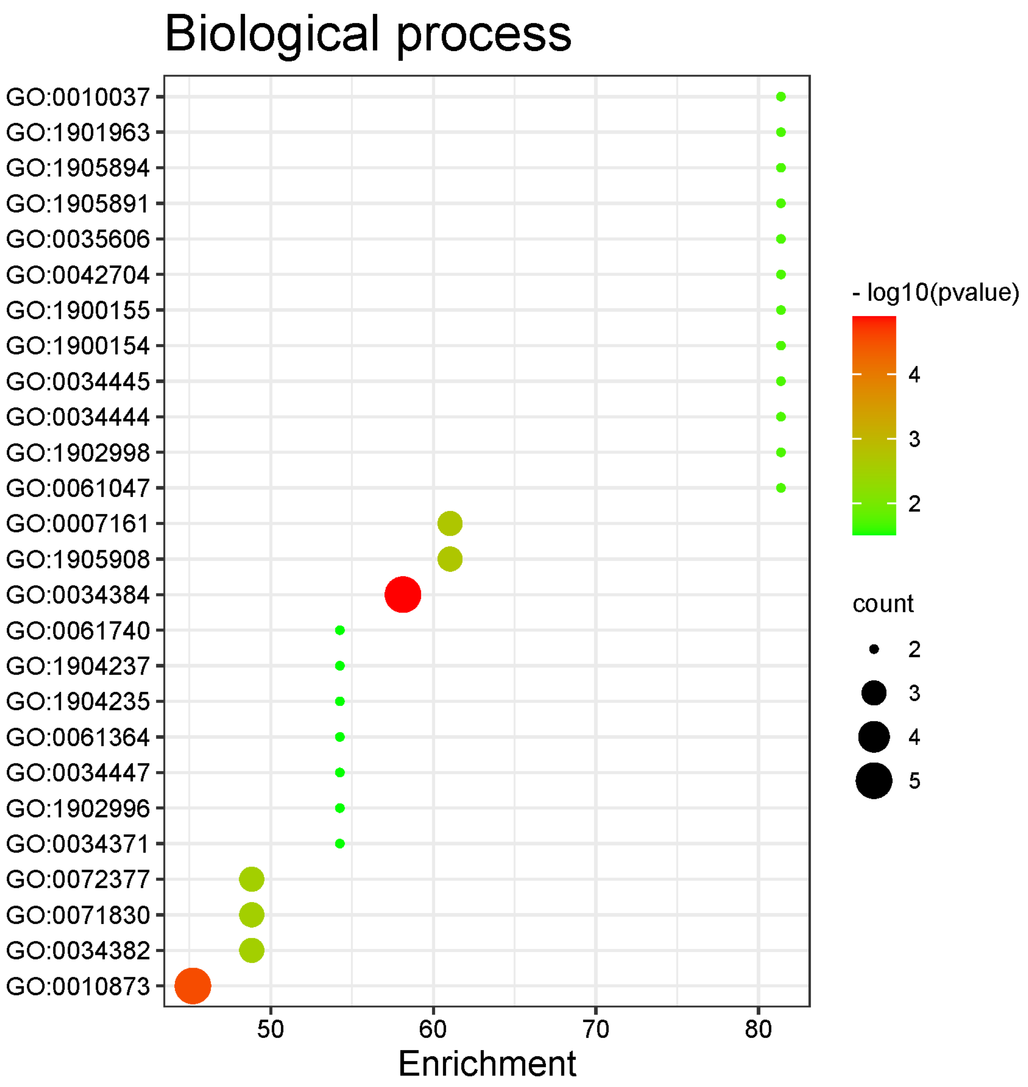

Supplementary figure S3. Representation of biological process GO categories with higher enrichment in bone ECM proteins of *Homo sapiens*.

GO:0010037, response to carbon dioxide; GO:1901963, regulation of cell proliferation involved in outflow tract morphogenesis; GO:1905894, regulation of cellular response to tunicamycin; GO:1905891, regulation of cellular response to thapsigargin; GO:0035606, peptidyl-cysteine S-trans-nitrosylation; GO:0042704, uterine wall breakdown; GO:1900155, negative regulation of bone trabecula formation; GO:1900154, regulation of bone trabecula formation; GO:0034445, negative regulation of plasma lipoprotein oxidation; GO:0034444, regulation of plasma lipoprotein oxidation; GO:1902998, positive regulation of neurofibrillary tangle assembly; GO:0061047, positive regulation of branching involved in lung morphogenesis; GO:0007161, calcium-independent cell-matrix adhesion; GO:1905908, positive regulation of amyloid fibril formation; GO:0034384, high-density lipoprotein particle clearance; GO:0061740, protein targeting to lysosome involved in chaperone-mediated autophagy; GO:1904237, positive regulation of substrate-dependent cell migration, cell attachment to substrate; GO:1904235, regulation of substrate-dependent cell migration, cell attachment to substrate; GO:0061364, apoptotic process involved in luteolysis; GO:0034447, very-low-density lipoprotein particle clearance; GO:1902996, regulation of neurofibrillary tangle assembly; GO:0034371, chylomicron remodeling; GO:0072377, blood coagulation, common pathway; GO:0071830, triglyceride-rich lipoprotein particle clearance; GO:0034382, chylomicron remnant clearance; and GO:0010873, positive regulation of cholesterol esterification.

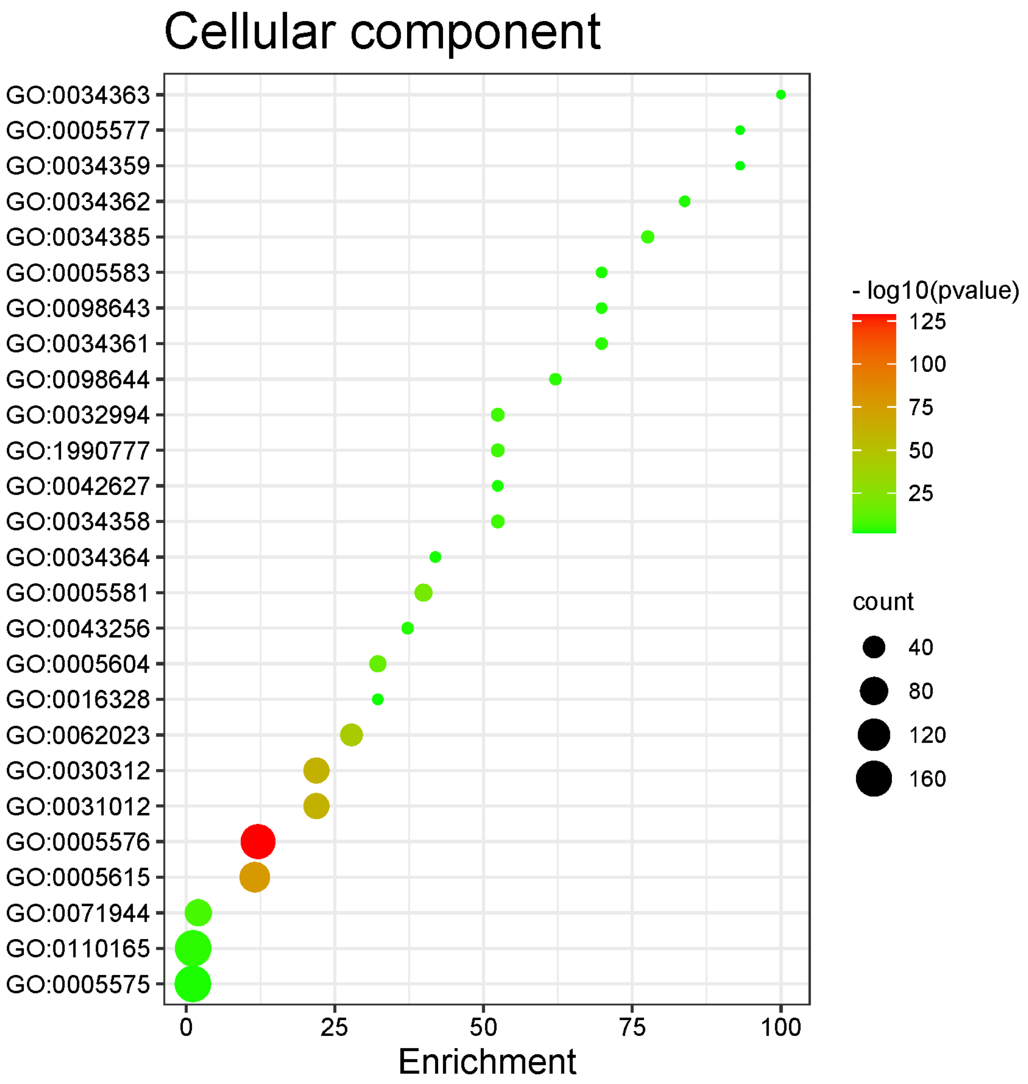

Supplementary figure S4. Representation of cellular components GO categories with higher enrichment in bone ECM proteins of *Danio rerio*.
GO:0034363, intermediate-density lipoprotein particle; GO:0005577, fibrinogen complex; GO:0034359, mature chylomicron; GO:0034362, low-density lipoprotein particle; GO:0034385, triglyceride-rich plasma lipoprotein particle; GO:0005583, fibrillar collagen trimer; GO:0098643, banded collagen fibril; GO:0034361, very-low-density lipoprotein particle; GO:0098644, complex of collagen trimers; GO:0032994, protein-lipid complex; GO:1990777, lipoprotein particle; GO:0042627, chylomicron; GO:0034358, plasma lipoprotein particle; GO:0034364, high-density lipoprotein particle; GO:0005581, collagen trimer; GO:0043256, laminin complex; GO:0005604, basement membrane; GO:0016328, lateral plasma membrane; GO:0062023, collagen-containing extracellular matrix; GO:0030312, external encapsulating structure; GO:0031012, extracellular matrix; GO:0005576, extracellular region; GO:0005615, extracellular space; GO:0071944, cell periphery; GO:0110165, cellular anatomical entity; and GO:0005575, cellular_component. Points with Enrichment 100 mean 100 or more.

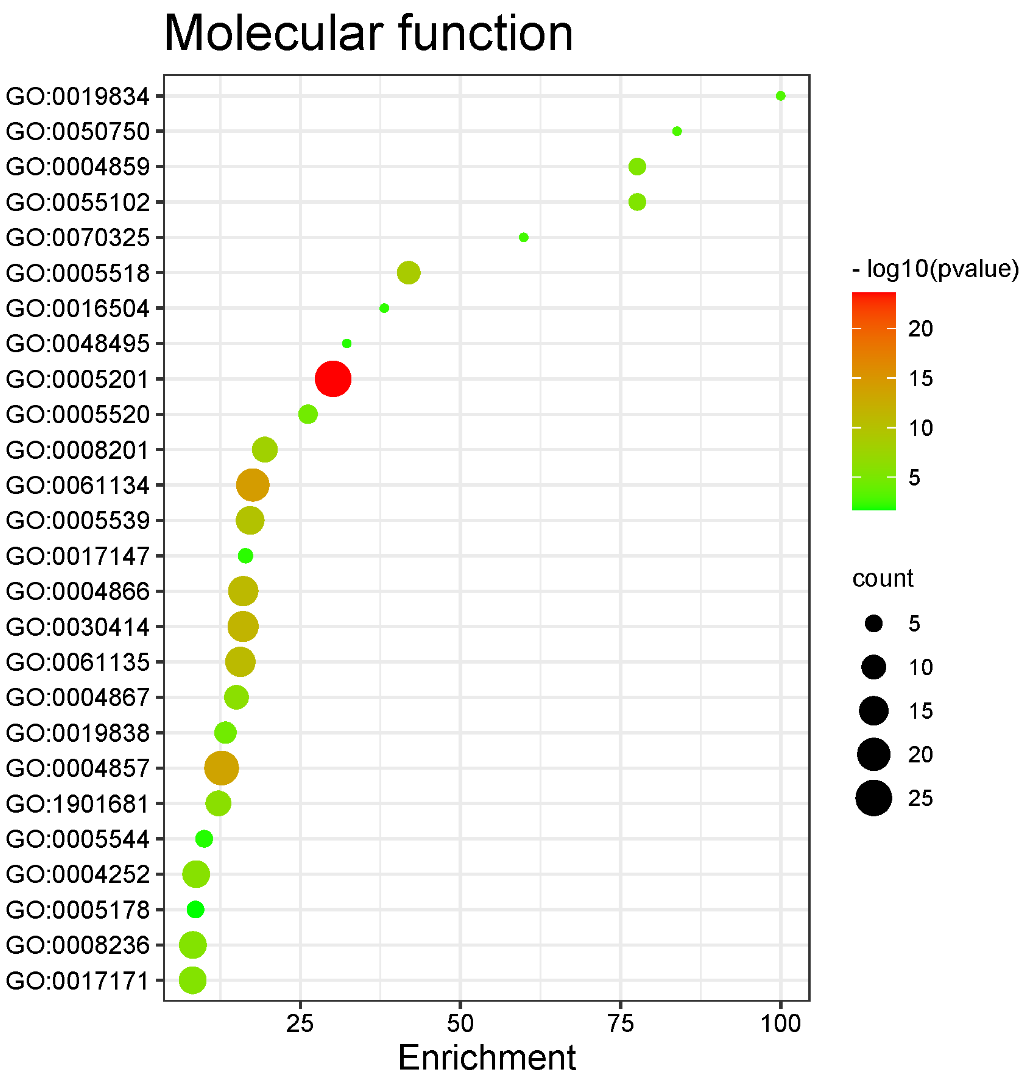

Supplementary figure S5. Representation of molecular function GO categories with higher enrichment in bone ECM proteins of *Danio rerio*.

GO:0019834, phospholipase A2 inhibitor activity; GO:0050750, low-density lipoprotein particle receptor binding; GO:0004859, phospholipase inhibitor activity; GO:0055102, lipase inhibitor activity; GO:0070325, lipoprotein particle receptor binding; GO:0005518, collagen binding; GO:0016504, peptidase activator activity; GO:0048495, Roundabout binding; GO:0005201, extracellular matrix structural constituent; GO:0005520, insulin-like growth factor binding; GO:0008201, heparin binding; GO:0061134, peptidase regulator activity; GO:0005539, glycosaminoglycan binding; GO:0017147, Wnt-protein binding; GO:0004866, endopeptidase inhibitor activity; GO:0030414, peptidase inhibitor activity; GO:0061135, endopeptidase regulator activity; GO:0004867, serine-type endopeptidase inhibitor activity; GO:0019838, growth factor binding; GO:0004857, enzyme inhibitor activity; GO:1901681, sulfur compound binding; GO:0005544, calcium-dependent phospholipid binding; GO:0004252, serine-type endopeptidase activity; GO:0005178, integrin binding; GO:0008236, serine-type peptidase activity; and GO:0017171, serine hydrolase activity. Points with Enrichment 100 mean 100 or more.

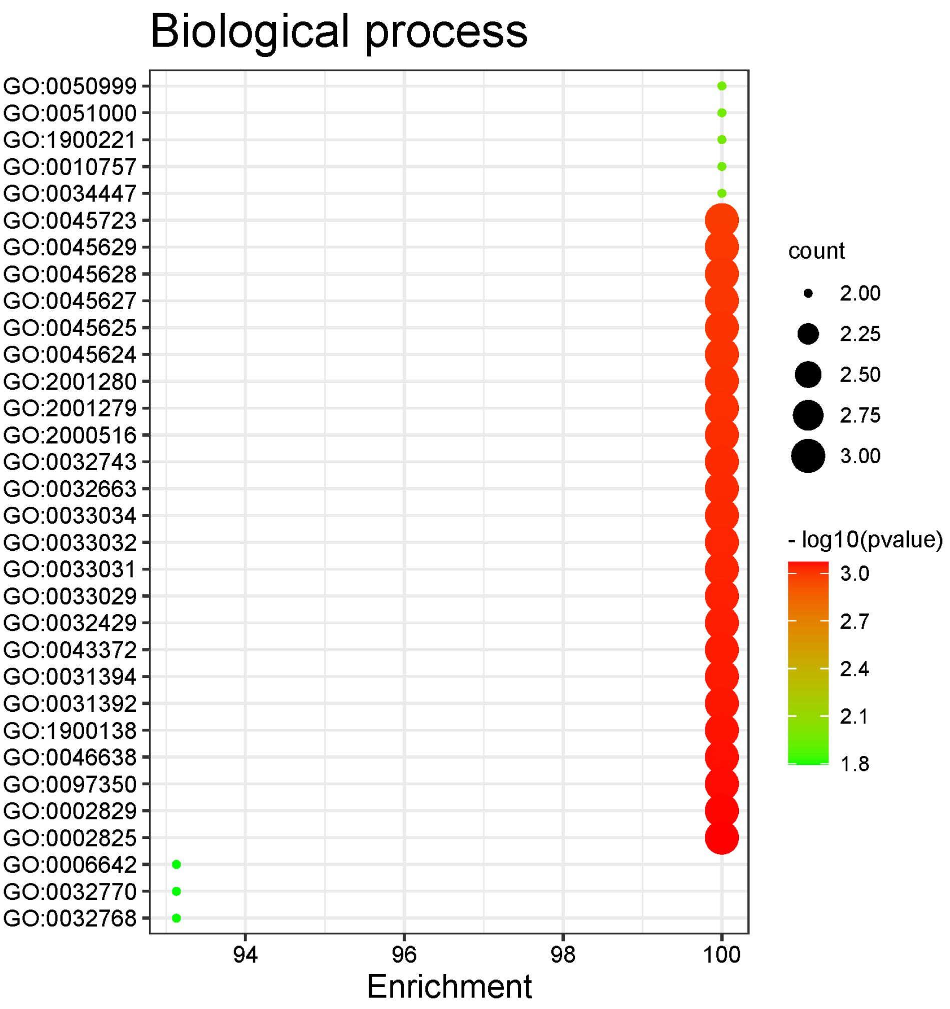

Supplementary figure S6. Representation of biological process GO categories with higher enrichment in bone ECM proteins of *Danio rerio*.

GO:0050999, regulation of nitric-oxide synthase activity; GO:0051000, positive regulation of nitric-oxide synthase activity; GO:1900221, regulation of amyloid-beta clearance; GO:0010757, negative regulation of plasminogen activation; GO:0034447, very-low-density lipoprotein particle clearance; GO:0045723, positive regulation of fatty acid biosynthetic process; GO:0045629, negative regulation of T-helper 2 cell differentiation; GO:0045628, regulation of T-helper 2 cell differentiation; GO:0045627, positive regulation of T-helper 1 cell differentiation; GO:0045625, regulation of T-helper 1 cell differentiation; GO:0045624, positive regulation of T-helper cell differentiation; GO:2001280, positive regulation of unsaturated fatty acid biosynthetic process; GO:2001279, regulation of unsaturated fatty acid biosynthetic process; GO:2000516, positive regulation of CD4-positive, alpha-beta T cell activation; GO:0032743, positive regulation of interleukin-2 production; GO:0032663, regulation of interleukin-2 production; GO:0033034, positive regulation of myeloid cell apoptotic process; GO:0033032, regulation of myeloid cell apoptotic process; GO:0033031, positive regulation of neutrophil apoptotic process; GO:0033029, regulation of neutrophil apoptotic process; GO:0032429, regulation of phospholipase A2 activity; GO:0043372, positive regulation of CD4-positive, alpha-beta T cell differentiation; GO:0031394, positive regulation of prostaglandin biosynthetic process; GO:0031392, regulation of prostaglandin biosynthetic process; GO:1900138, negative regulation of phospholipase A2 activity; GO:0046638, positive regulation of alpha-beta T cell differentiation; GO:0097350, neutrophil clearance; GO:0002829, negative regulation of type 2 immune response; GO:0002825, regulation of T-helper 1 type immune response; GO:0006642, triglyceride mobilization; GO:0032770, positive regulation of monooxygenase activity; and GO:0032768, regulation of monooxygenase activity. Points with Enrichment 100 mean 100 or more.

SUPPLEMENTARY TABLES

Supplementary table S1. Proteins used as seed to build the Phylobone database.

PB ID	Uniprot *D. rerio*	Gene name *D. rerio*	Uniprot *H. sapiens*	Gene name *H. sapiens*	Uniprot C. nippon	Gene Name C. nippon	Ref.
**PB0001**	A5WWI5	ahsg	P02765	AHSG			[1]
**PB0002**	B0UYS1	b2m	P61769	B2M			[1]
**PB0003**	E7FAN5	f2	P00734	F2			[1]
**PB0004**	Q6YND0	mgp	P08493	MGP			[1]
**PB0005**	B7ZDB5	bgnb	P21810	BGN		BGN	[1]
**PB0005**	Q6GMI5	bgna	P21810	BGN		BGN	[1]
**PB0006**	Q6PVV8	sparc	P09486	SPARC		SPARC	[1]
**PB0007**	F1Q7R2	chad	O15335	CHAD			[1]
**PB0008**	F1R6R2	APCS	P02743	APCS			[1]
**PB0009**	Q66I20	serpinf1	P36955	SERPINF1			[1]
**PB0010**			P02792	FTL			[1]
**PB0011**			P04406	GAPDH			[1]
**PB0012**			P06702	S100A9			[1]
**PB0013**			P08311	CTSG			[1]
**PB0014**			P24158	PRTN3			[1]
**PB0015**			P28300	LOX		LOX	[1]
**PB0016**			P62805	HIST1H4A			[1]
**PB0017**			P69905	HBA1			[1]
**PB0018**	Q6IQX2	col1a2	P08123	COL1A2		COL1A2	[1]
**PB0019**	F1QDL1	col1a1b	P02452	COL1A1		COL1A1	[1]
**PB0019**	F1QJC9	col1a1a	P02452	COL1A1		COL1A1	[1]
**PB0020**	A0JML7	adpgk	Q9BRR6	ADPGK			[1]
**PB0021**	A1IGX5	fibin	Q8TAL6	FIBIN			[1]
**PB0022**	A1L1V4	loxl2b	Q9Y4K0	LOXL2			[1]
**PB0023**	A2AVJ4	bmp3	P12645	BMP3			[1]
**PB0024**	A2CEN1	efemp2a	Q6FH22	EFEMP2			[1]
**PB0025**	A2CEW3	fn1b	P02751	FN1		FN1	[1]
**PB0026**	A2RRT1	mfge8b	Q08431	MFGE8			[1]
**PB0027**	A2RUX5	col11a1a	P12107	COL11A1		COL11A1	[1]
**PB0027**	D6MUD3	col11a1b	P12107	COL11A1		COL11A1	[1]
**PB0028**	A3KNP6	sfrp1b	Q8N474	SFRP1			[1]
**PB0028**	Q7T2K9	sfrp1a	Q8N474	SFRP1			[1]
**PB0029**	A3KQQ9	scg3	Q8WXD2	SCG3			[1]
**PB0030**	A5PMP6	c3b.1	P01024	C3			[1]
**PB0030**	B8JKW4	c3a	P01024	C3			[1]
**PB0030**	F1QX13	c3c	P01024	C3			[1]
**PB0031**	A5WWJ4	matn1	P21941	MATN1			[1]
**PB0032**	A7MCK2	hsd17b12b	Q53GQ0	HSD17B12			[1]
**PB0033**	A8E5G1	anxa2b	P07355	ANXA2			[1]
**PB0034**	E2FHP6	cfhl2	P36980	CFHR2			[1]
**PB0035**	A8KC28	proc	P04070	PROC			[1]
**PB0035**	B8JLG2	proc	P04070	PROC			[1]
**PB0036**	A9JRB3	htra1b	Q92743	HTRA1			[1]
**PB0036**	Q6GMI0	htra1a	Q92743	HTRA1			[1]
**PB0037**	B0JZL7	enpp1	P22413	ENPP1			[1]
**PB0038**	B0S525	igfbp5b	P24593	IGFBP5			[1]
**PB0039**	P58239	lect1	O75829	LECT1			[1]

**PB0040**	B0S5W0	col8a1a	P27658	COL8A1		1
**PB0041**	B0S5X0	epyc	Q99645	EPYC		1
**PB0042**	B0S6C1	tfpia	P48307	TFPI2		1
**PB0043**	B0S6K5	tnn	Q9UQP3	TNN		1
**PB0044**	B0UXF7	col4a5	P29400	COL4A5		1
**PB0045**	B0UXN0	qsox1	O00391	QSOX1		1
**PB0046**	B0UXR7	olfml2a	Q68BL7	OLFML2A		1
**PB0047**	B3DFP9	apoa2	P02652	APOA2		1
**PB0048**	B3DFV8	coch	O43405	COCH		1
**PB0049**	B3DHC5	apoa4b.2	P06727	APOA4		1
**PB0050**	B3DJG2	pcolce (2/2)	Q15113	PCOLCE		1
**PB0050**	F1QUX0	pcolce (1/2)	Q15113	PCOLCE		1
**PB0051**	E7FG81	col12a1	Q99715	COL12A1		1
**PB0051**	E7FG81	col12a1	Q99715	COL12A1		1
**PB0052**	B7SDQ7	ccdc80	Q76M96	CCDC80		1
**PB0053**	B7ZDA6	lamb2	P55268	LAMB2		1
**PB0053**	F1QKX8	lamb2l	P55268	LAMB2P1		1
**PB0054**	B8A4T6	col9a1	P20849	COL9A1		1
**PB0054**	F1QTQ2	col9a1	P20849	COL9A1		1
**PB0055**	B8JHU4	actn1	P12814	ACTN1		1
**PB0056**	E9QIM2	sod3b	P08294	SOD3		1
**PB0057**	B8JL29	hmgb2a	P26583	HMGB2		1
**PB0057**	Q66IB6	hmgb2b	P26583	HMGB2		1
**PB0058**	B8JL43	tfa	P02787	TF		1
**PB0059**	B8JLZ3	anxa1b	P04083	ANXA1		1
**PB0059**	Q804H0	anxa1c	P04083	ANXA1		1
**PB0059**	Q804H2	anxa1a	P04083	ANXA1		1
**PB0060**	B8XY56	rnaset2	O00584	RNASET2		1
**PB0061**	C1IHU8	itln1	Q8WWA0	ITLN1		1
**PB0062**	E7F7K2	c1qtnf9	B2RNN3	C1QTNF9B		1
**PB0063**	E7EXE8	tnxb	P22105	TNXB		1
**PB0064**	E7EYJ8	clec3ba	P05452	CLEC3B		1
**PB0065**	E7F1S4	thbs2b	P35442	THBS2		1
**PB0065**	F8W5Z7	thbs2a	P35442	THBS2		1
**PB0066**	E7F2E3	mfi2	P08582	MFI2		1
**PB0067**	E7F301	f9a	P00740	F9		1
**PB0067**	Q1RLV2	f9b	P00740	F9		1
**PB0068**	E7F537	acana	P16112	ACAN		1
**PB0068**	F1R511	acanb	P16112	ACAN		1
**PB0069**	E7F5I3	postna	Q15063	POSTN	POSTN	1
**PB0069**	Q75U66	postnb	Q15063	POSTN	POSTN	1
**PB0070**	X1WE42	nid2	Q14112	NID2	NID2	1
**PB0071**	B8A4S4	col17a1a	Q9UMD9	COL17A1		1
**PB0072**	E7F829	aebp1	Q8IUX7	AEBP1		1
**PB0073**	A0A286Y9F3	otos	Q8NHW6	OTOS		1
**PB0074**	E7F8X0	SRPX2	O60687	SRPX2		1
**PB0075**	F1RAG3	nid1a	P14543	NID1	NID1	1
**PB0076**	E7FB76	col21a1	Q96P44	COL21A1		1
**PB0077**	E7FBD3	apob	P04114	APOB		1
**PB0077**	Q5TZ29	apobb	P04114	APOB		1
**PB0078**	E7FBW8	hapln1b	P10915	HAPLN1		1
**PB0078**	Q1LXE1	hapln1a	P10915	HAPLN1		1
**PB0079**	D6MUD6	col5a3a	P25940	COL5A3		1
**PB0079**	D6MUD7	col5a3b	P25940	COL5A3		1

**PB0080**	E7FCV2	c5	P01031	C5		1
**PB0081**	E7FCV8	col6a2	P12110	COL6A2	COL6A2	1
**PB0082**	E7FDK4	podn	Q7Z5L7	PODN		1
**PB0083**	X1WG18	muc5b	Q9HC84	MUC5B		1
**PB0084**	E7FE47	col5a2b	P05997	COL5A2	COL5A2	1
**PB0085**	E7FE90	fetub	Q9UGM5	FETUB		1
**PB0086**	A0A0R4IKF0	apoa1b	P02647	APOA1		1
**PB0087**	E9QDI1	apoc1l	P02654	APOC1		1
**PB0088**	E9QEQ1	apoc2	P02655	APOC2		1
**PB0089**	E9QG22	spp2	Q13103	SPP2		1
**PB0090**	F1Q4X1	col6a3	P12111	COL6A3	COL6A3	1
**PB0091**	K7DYI0	col16a1	Q07092	COL16A1		1
**PB0092**	F1Q5B5	alpl	P05186	ALPL		1
**PB0093**	A2AR69	itih6	Q6UXX5	ITIH6		1
**PB0094**	F1Q5N7	cilp2	Q8IUL8	CILP2		1
**PB0095**	F1Q5S2	proza	P22891	PROZ		1
**PB0095**	F1QZU6	proz	P22891	PROZ		1
**PB0096**	F1Q6P3	col6a1	P12109	COL6A1	COL6A1	1
**PB0097**			Q8N2E2	VWDE		1
**PB0098**	F1QG51	fmoda	Q06828	FMOD		1
**PB0099**	F1Q775	cilp	O75339	CILP		1
**PB0100**	F1Q8A0	ctsk	P43235	CTSK		1
**PB0101**	X1WEZ4	col6a4a	A6NMZ7	col6a6		1
**PB0102**	F1Q9F4	spp1	P10451	SPP1		1
**PB0103**	Q7ZV46	metrnl	Q641Q3	METRNL		1
**PB0104**	A0A286Y830	emid1	Q96A84	EMID1		1
**PB0104**	F1QR06	emid1	Q96A84	EMID1		1
**PB0105**	Q8JHV6	lamb4	A4D0S4	LAMB4		1
**PB0106**	F1QBE9	ccl25b	O15444	CCL25		1
**PB0107**	F1QC76	mmp9	P14780	MMP9		1
**PB0108**	F1QEE7	thbs1b	P07996	THBS1	THBS1	1
**PB0109**	F1QFH8	apom	O95445	APOM		1
**PB0110**	F1QFP3	f7	P08709	F7		1
**PB0111**	F1QFX6	ltbp3	Q9NS15	LTBP3		1
**PB0112**	Q71G59	mmp13b	P45452	MMP13		1
**PB0113**	F1QG79	col4a1	P02462	COL4A1		1
**PB0114**	F1QGG9	gas6	Q14393	GAS6		1
**PB0115**	F1QHI8	pdgfrl	Q15198	PDGFRL		1
**PB0116**			P04004	VTN	VTN	1
**PB0117**	F1QIJ3	lamc2	Q13753	LAMC2		1
**PB0118**	F1QJB3	c2	P06681	C2		1
**PB0119**	F1QJG6	sfrp5	Q5T4F7	SFRP5		1
**PB0120**	F1QK29	pros1	P07225	PROS1		1
**PB0121**	F1QK57	tecta	O75443	TECTA		1
**PB0122**	F1QKL1	thbs3a	P49746	THBS3		1
**PB0123**	F1QKW3	lama5	O15230	LAMA5		1
**PB0124**	F1QLC3	f10	P00742	F10		1
**PB0125**	F1QLH9	nucb1	Q02818	NUCB1		1
**PB0126**	F1QLT3	fgfbp2	Q9BYJ0	FGFBP2		1
**PB0126**	F1R8L1	fgfbp2	Q9BYJ0	FGFBP2		1
**PB0127**	F1QLW6	ogn (2/2)	P20774	OGN		1
**PB0127**	Q1LV51	ogn	P20774	OGN		1
**PB0128**	Q8JHV7	lamb1a	P07942	LAMB1	LAMB1	1
**PB0128**	F1REL9	lamb1b	P07942	LAMB1	LAMB1	1

**PB0129**	L0S5L0	col2a1b	P02458	COL2A1		[1]
**PB0129**	Q2LDA1	col2a1a	P02458	COL2A1		[1]
**PB0130**	F1QPX0	ndnf	Q8TB73	NDNF		[1]
**PB0131**	F1QQE4	slit2	O94813	SLIT2		[1]
**PB0132**	F1QRB8	serp1	P05121	SERPINE1		[1]
**PB0133**	B3DKC3	mfap2	P55001	MFAP2		[1]
**PB0134**	F1QTG2	vwa2	Q5GFL6	VWA2		[1]
**PB0135**	A0A2R8Q7H6	lama3	Q16787	LAMA3		[1]
**PB0136**	F1QW52	habp2	Q14520	HABP2		[1]
**PB0137**	F1QXD5	col10a1	Q03692	COL10A1		[1]
**PB0138**	F1QY29	prelp	P51888	PRELP		[1]
**PB0139**	F1QYE2	tnc	P24821	TNC	TNC	[1]
**PB0140**	F1QYN1	col4a6	Q14031	COL4A6		[1]
**PB0141**	F1QYU0	lama4	Q16363	LAMA4	LAMA4	[1]
**PB0142**	F1R074	agrn	O00468	AGRN		[1]
**PB0143**	F1R0N0	lrrc17	Q8N6Y2	LRRC17		[1]
**PB0144**	F1R147	pla2g12b	Q9BX93	PLA2G12B		[1]
**PB0145**	F1R1F1	col9a3	Q14050	COL9A3		[1]
**PB0146**	F1R1P9	thbs4b	P35443	THBS4		[1]
**PB0147**	A0A0H2UKP2	col4a2	P08572	COL4A2		[1]
**PB0148**	F1R2S9	ecm2	O94769	ECM2	ECM2	[1]
**PB0149**	F1R2W1	dhrs13a.3	Q6UX07	DHRS13		[1]
**PB0150**	F1R2Z9	spon1b	Q9HCB6	SPON1		[1]
**PB0151**	D6MUD4	col11a2	P13942	COL11A2		[1]
**PB0152**	F1R5K7	hspg2	P98160	HSPG2	HSPG2	[1]
**PB0153**	F1R5V4	col4a3	Q01955	COL4A3		[1]
**PB0154**	F1R5Z2	fndc1	Q4ZHG4	FNDC1		[1]
**PB0155**	Q6IQQ7	lum	P51884	LUM	LUM	[1]
**PB0156**			P35555	FBN1		[1]
**PB0157**	F1R774	pcolce2b	Q9UKZ9	PCOLCE2		[1]
**PB0158**	Q5KQU0	angptl1	O95841	ANGPTL1		[1]
**PB0158**	Q5RH53	angptl1	O95841	ANGPTL1		[1]
**PB0159**	F1R886	cfb	P00751	CFB		[1]
**PB0160**	Q7SZV4	tgfb2	P61812	TGFB2		[1]
**PB0161**	E9QI62	flna	P21333	FLNA		[1]
**PB0162**	A0A0G2L5F9	cfi	P05156	CFI		[1]
**PB0163**	F1R9Y1	col14a1	Q05707	COL14A1	COL14A1	[1]
**PB0164**	A6H8K2	bmper	Q8N8U9	BMPER		[1]
**PB0165**	F1RAP6	serpinf2b	P08697	SERPINF2		[1]
**PB0166**	Q9PWH3	dkk1a	O94907	DKK1		[1]
**PB0167**	F6P711	cpz	Q66K79	CPZ		[1]
**PB0168**	F1RCV3	slit3	O75094	SLIT3		[1]
**PB0169**	F1RDU8	FCGBP	Q9Y6R7	FCGBP		[1]
**PB0170**	A0A0G2KQ62	fbn2b	Q75N90	FBN3		[1]
**PB0171**	F1REI0	htra3	P83110	HTRA3		[1]
**PB0172**	A0A0R4IF38	esm1	Q9NQ30	ESM1		[1]
**PB0173**	F1REM3	bmp1a	P13497	BMP1		[1]
**PB0174**	Q8AV83	try	P07477	PRSS1		[1]
**PB0175**	Q5SPJ4	serpina1	P01009	SERPINA1		[1]
**PB0176**	Q503K1	tgfbi	Q15582	TGFBI	TGFBI	[1]
**PB0177**	F2Z4S3	tcn2l	P20062	TCN2		[1]
**PB0178**	O42222	mstnb	O14793	MSTN		[1]
**PB0179**	O42364	apoeb	P02649	APOE		[1]
**PB0179**	Q503V2	apoea	P02649	APOE		[1]

**PB0180**	Q15JE7	optc	Q9UBM4	OPTC		[1]
**PB0181**	Q1L8M3	fgf23	Q9GZV9	FGF23		[1]
**PB0182**	Q1L8P0	crtap	O75718	CRTAP		[1]
**PB0183**	Q1LUC6	tinagl1	Q9GZM7	TINAGL1		[1]
**PB0184**	Q1LVF0	lamc1	P11047	LAMC1	LAMC1	[1]
**PB0185**	Q1LXA7	aspn	Q9BXN1	ASPN		[1]
**PB0186**	Q1LXU2	col9a2	Q14055	COL9A2		[1]
**PB0187**	Q1LYJ7	kng1	P01042	KNG1		[1]
**PB0188**	Q1RLX2	timp2a	P16035	TIMP2		[1]
**PB0189**	Q24JW2	lyz	P61626	LYZ		[1]
**PB0190**	Q29RB4	olfml3a	Q9NRN5	OLFML3		[1]
**PB0191**	Q29RF0	dpt	Q07507	DPT		[1]
**PB0192**	Q2PMI2	fstl1b	Q12841	FSTL1		[1]
**PB0193**	Q5F0G5	myoc	Q99972	MYOC		[1]
**PB0194**	Q5NJJ5	matn4	O95460	MATN4		[1]
**PB0195**	Q5NJJ7	matn3b	O15232	MATN3		[1]
**PB0195**	Q68EK6	matn3a	O15232	MATN3		[1]
**PB0196**	Q5RI33	ctgfa	P29279	CTGF		[1]
**PB0197**	Q5RI43	kera	O60938	KERA		[1]
**PB0198**	Q5RI45	dcn	P07585	DCN	DCN	[1]
**PB0199**	Q5RIH8	serpina10a	Q9UK55	SERPINA10		[1]
**PB0200**	Q5SPR2	clu	P10909	CLU		[1]
**PB0201**	Q66I23	tgfb3	P10600	TGFB3		[1]
**PB0202**	Q6AXL0	cthrc1a	Q96CG8	CTHRC1		[1]
**PB0203**	Q6AZC0	cela1	Q9UNI1	CELA1		[1]
**PB0204**	Q6DG10	mmp2	P08253	MMP2	MMP2	[1]
**PB0205**	Q6IQP3	calua	O43852	CALU		[1]
**PB0206**	Q6NWB6	ucmab	Q8WVF2	UCMA		[1]
**PB0207**	Q6NXA5	pon2	Q15165	PON2		[1]
**PB0208**	Q6NYE1	fgb	P02675	FGB	FGB	[1]
**PB0209**	Q6NYR4	hspa8	P11142	HSPA8		[1]
**PB0210**			P78380	OLR1		[1]
**PB0211**	Q6P0V8	anxa5b	P08758	ANXA5		[1]
**PB0212**	Q6PC07	rbp4l	P02753	RBP4		[1]
**PB0213**	Q6T937	grem1a	O60565	GREM1		[1]
**PB0214**	Q7ZVG7	fgg	P02679	FGG	FGG	[1]
**PB0215**	Q7ZVL5	serpine2	P07093	SERPINE2		[1]
**PB0216**	Q803H7	itm2ba	Q9Y287	ITM2B		[1]
**PB0217**	Q8QGV5	ptgdsb	Q6UWW0	LCN15		[1]
**PB0218**	Q9DDG2	mdkb	P21741	MDK		[1]
**PB0218**	Q9W767	mdka	P21741	MDK		[1]
**PB0219**	Q9PTH3	igfbp2a	P18065	IGFBP2		[1]
**PB0220**	Q9W6E0	frzb	Q92765	FRZB		[1]
**PB0221**	Q9YHV4	fsta	P19883	FST		
**PB0222**			P01023	A2M	A2M	[2]
**PB0223**			P05067	APP	APP	[2]
**PB0224**			P40121	CAPG	CAPG	[2]
**PB0225**			P07384	CAPN1	CAPN1	[2]
**PB0226**			P17655	CAPN2	CAPN2	[2]
**PB0227**			P04632	CAPNS1	CAPNS1	[2]
**PB0228**			P16070	CD44	CD44	[2]
**PB0229**			P39060	COL18A1	COL18A1	[2]
**PB0230**			P02461	COL3A1	COL3A1	[2]
**PB0231**			P23142	FBLN1	FBLN1	[2]

**PB0232**	P02671	FGA		FGA	2
**PB0233**	P06396	GSN		GSN	2
**PB0234**	P08648	ITGA5		ITGA5	2
**PB0235**	P06756	ITGAV		ITGAV	2
**PB0236**	P05556	ITGB1		ITGB1	2
**PB0237**	P13796	LCP1		LCP1	2
**PB0238**	P00747	PLG		PLG	2
**PB0239**	Q13162	PRDX4		PRDX4	2
**PB0240**	P36952	SERPINB5		SERPINB5	2
**PB0241**	A1X283	SH3PXD2B		SH3PXD2B	2
**PB0242**	P02766	TTR		TTR	2
**PB0243**	P13611	VCAN		VCAN	2
**PB0244**	Q15942	ZYX		ZYX	2
**PB0245**	P35222	CTNNB1		CTNNB1	2
**PB0246**	P63000	RAC1		RAC1	2
**PB0247**	Q96AC1	FERMT2		FERMT2	2
**PB0248**	P18206	VCL		VCL	2
**PB0249**	P21815	IBSP	E7D7Z0	BSPII	3
**PB0250**	Q99217	AMELX			4
**PB0251**	P12644	BMP4			5
**PB0252**	Q2MKA7	RSPO1			6
**PB0253**	Q6UXX9	RSPO2			6
**PB0254**	Q9BXY4	RSPO3			6
**PB0255**	Q2I0M5	RSPO4			6

Supplementary table S2. List of selected species included in the Phylobone database.

N	Species		Taxonomic group		Proteins
1	*Homo sapiens (*)*	Human	Vertebrates	Primates	255
2	*Pan troglodytes*	Chimpanzee	Vertebrates	Primates	255
3	*Pan paniscus*	Pygmy chimpanzee	Vertebrates	Primates	255
4	*Gorilla gorilla*	Western gorilla	Vertebrates	Primates	247
5	*Pongo abelii*	Sumatran orangutan	Vertebrates	Primates	255
6	*Oryctolagus cuniculus*	Rabbit	Vertebrates	Rabbits	244
7	*Mus musculus*	Mouse	Vertebrates	Rodents	253
8	*Rattus norvegicus*	Rat	Vertebrates	Rodents	253
9	*Felis catus*	Cat	Vertebrates	Carnivores	250
10	*Canis lupus familiaris*	Dog	Vertebrates	Carnivores	250
11	*Cervus elaphus*	Red deer	Vertebrates	Even-toed ungulates	168
12	*Cervus hanglu*	Central Asian red deer	Vertebrates	Even-toed ungulates	171
13	*Cervus canadensis*	Elk	Vertebrates	Even-toed ungulates	245
14	*Odocoileus virginianus*	White-tailed deer	Vertebrates	Even-toed ungulates	243
15	*Muntiacus muntjak*	Indian muntjac	Vertebrates	Even-toed ungulates	202
16	*Muntiacus reevesi*	Chinese muntjac	Vertebrates	Even-toed ungulates	186
17	*Capra hircus*	Goat	Vertebrates	Even-toed ungulates	251
18	*Ovis aries*	Sheep	Vertebrates	Even-toed ungulates	251
19	*Bos taurus*	Bull	Vertebrates	Even-toed ungulates	253
20	*Sus scrofa*	Pig	Vertebrates	Even-toed ungulates	251
21	*Alligator mississippiensis*	American alligator	Vertebrates	Reptiles	243
22	*Alligator sinensis*	Chinese alligator	Vertebrates	Reptiles	238

23	*Columba livia*	Rock pigeon	Vertebrates	Birds	224
24	*Gallus gallus*	Chicken	Vertebrates	Birds	244
25	*Meleagris gallopavo*	Turkey	Vertebrates	Birds	216
26	*Xenopus laevis*	African clawed frog	Vertebrates	Frogs and toads	248
27	*Rana temporaria*	common frog	Vertebrates	Frogs and toads	245
28	*Danio rerio (*)*	Zebrafish	Vertebrates	Bony fishes	251
29	*Nothobranchius furzeri*	Turquoise killifish	Vertebrates	Bony fishes	245
30	*Oryzias latipes*	Japanese medaka	Vertebrates	Bony fishes	246
31	*Sparus aurata*	Gilthead seabream	Vertebrates	Bony fishes	249
32	*Styela clava*	Asian tunicate	Chordata	Tunicata	188
33	*Patiria miniata*	Bat star	Invertebrates	Echinoderm	179
34	*Strongylocentrotus purpuratus*	Purple sea urchin	Invertebrates	Echinoderm	182
35	*Drosophila melanogaster*	Fruit fly	Invertebrates	Insects	135
36	*Apis mellifera*	Honey bee	Invertebrates	Insects	151
37	*Caenorhabditis elegans*	Worm	Invertebrates	Nematoda	115
38	*Sepia pharaonis*	Cuttlefish	Invertebrates	Cephalopoda	120
39	*Actinia tenebrosa*	Anemone	Invertebrates	Cnidaria	158

(*) Used as seed to identify proteins in other organisms

Supplementary table S3. Phyletic patterns.

Groups	Pr	Ra	Ro	Ca	De	Bo	Pi	Re	Bi	Am	Fi	Tu	Ec	In	Ne	Ce	Cn
N	5	1	2	2	6	3	1	2	3	2	4	1	2	2	1	1	1
PB0001	1	1	1	1	1	1	1	1	1	1	1	0	0	0	0	0	0
PB0002	1	1	1	1	1	1	1	1	1	1	1	0	0	0	0	0	0
PB0003	1	1	1	1	1	1	1	1	1	1	1	1	1	1	1	1	1
PB0004	1	1	1	1	1	1	1	1	1	1	1	0	0	0	0	0	0
PB0005	1	1	1	1	1	1	1	1	1	1	1	1	1	1	1	1	0
PB0006	1	1	1	1	1	1	1	1	1	1	1	1	1	1	1	0	1
PB0007	1	1	1	1	1	1	1	1	1	1	1	1	1	1	1	1	1
PB0008	1	1	1	1	1	1	1	1	1	1	1	1	1	1	0	0	1
PB0009	1	1	1	1	1	1	1	1	1	1	1	1	1	1	1	0	1
PB0010	1	1	1	1	1	1	1	1	1	1	1	1	1	1	1	1	1
PB0011	1	1	1	1	1	1	1	1	1	1	1	1	1	1	1	1	1
PB0012	1	1	1	1	1	1	1	1	1	1	1	0	0	0	0	0	0
PB0013	1	1	1	1	1	1	1	1	1	1	1	1	1	1	1	1	1
PB0014	1	1	1	1	1	1	1	1	1	1	1	1	1	1	1	1	1
PB0015	1	1	1	1	1	1	1	1	1	1	1	1	1	1	0	1	1
PB0016	1	1	1	1	1	1	1	1	1	1	1	1	1	1	1	0	1
PB0017	1	1	1	1	1	1	1	1	1	1	1	0	0	0	0	0	0
PB0018	1	1	1	1	1	1	1	1	1	1	1	1	1	1	0	1	1
PB0019	1	1	1	1	1	1	1	1	1	1	1	1	1	1	0	1	1
PB0020	1	1	1	1	1	1	1	1	1	1	1	1	1	1	1	1	1
PB0021	1	1	1	1	1	1	1	0	1	1	1	0	0	0	0	0	0
PB0022	1	1	1	1	1	1	1	1	1	1	1	1	1	1	0	1	1
PB0023	1	1	1	1	1	1	1	1	1	1	1	1	1	1	1	1	1
PB0024	1	1	1	1	1	1	1	1	1	1	1	1	1	1	1	1	1
PB0025	1	1	1	1	1	1	1	1	1	1	1	0	0	0	0	0	0
PB0026	1	1	1	1	1	1	1	1	1	1	1	1	1	0	0	1	0
PB0027	1	1	1	1	1	1	1	1	1	1	1	1	1	1	1	1	1
PB0028	1	1	1	1	1	1	1	1	1	1	1	1	1	1	1	1	1
PB0029	1	1	1	1	1	1	1	1	1	1	1	0	0	0	0	0	0

PB0030	1	1	1	1	1	1	1	1	1	1	1	1	1	1	0	1	1
PB0031	1	1	1	1	1	1	1	1	1	1	1	0	0	0	0	0	0
PB0032	1	1	1	1	1	1	1	1	1	1	1	1	1	1	1	1	1
PB0033	1	1	1	1	1	1	1	1	1	1	1	1	1	1	1	1	1
PB0034	1	1	1	1	1	1	1	1	1	1	1	0	1	0	0	0	1
PB0035	1	1	1	1	1	1	1	1	1	1	1	0	1	1	1	1	1
PB0036	1	1	1	1	1	1	1	1	1	1	1	1	1	1	0	1	0
PB0037	1	1	1	1	1	1	1	1	1	1	1	1	1	1	1	1	1
PB0038	1	1	1	1	1	1	1	1	1	1	1	1	0	0	0	0	0
PB0039	1	1	1	1	1	1	1	1	1	1	1	1	0	0	0	0	0
PB0040	1	1	1	1	1	1	1	1	1	1	1	0	1	0	0	0	0
PB0041	1	1	1	1	1	1	1	1	1	1	1	0	1	0	0	0	0
PB0042	1	1	1	1	1	1	1	1	1	1	1	0	0	0	0	0	1
PB0043	1	1	1	1	1	1	1	1	1	1	1	1	1	1	1	1	1
PB0044	1	1	0	1	1	1	1	1	1	1	1	1	1	1	1	1	1
PB0045	1	1	1	1	1	1	1	1	1	1	1	1	1	1	1	1	1
PB0046	1	1	1	1	1	1	1	1	1	1	1	1	1	1	1	0	0
PB0047	1	1	1	1	1	1	1	1	1	0	1	0	0	0	0	0	0
PB0048	1	1	1	1	1	1	1	1	1	1	1	0	0	0	1	0	0
PB0049	1	1	1	1	1	1	1	1	1	1	1	0	0	0	0	0	0
PB0050	1	1	1	1	1	1	1	1	1	1	1	1	1	1	1	1	1
PB0051	1	1	1	1	1	1	1	1	1	1	1	1	1	1	0	1	1
PB0052	1	1	1	1	1	1	1	1	1	1	1	0	0	0	0	0	0
PB0053	1	1	1	1	1	1	1	1	1	1	1	1	1	1	1	1	1
PB0054	1	1	1	1	1	1	1	1	1	1	1	1	1	1	1	1	1
PB0055	1	1	1	1	1	1	1	1	1	1	1	1	1	1	1	0	1
PB0056	1	1	1	1	1	1	1	1	1	1	1	1	1	1	1	1	1
PB0057	1	1	1	1	1	1	1	1	1	1	1	1	1	1	1	1	1
PB0058	1	1	1	1	1	1	1	1	1	1	1	1	1	1	0	1	1
PB0059	1	1	1	1	1	1	1	1	1	1	1	1	1	1	1	1	1
PB0060	1	1	1	1	1	1	1	1	1	1	1	1	1	1	1	1	1
PB0061	1	1	1	0	1	1	1	1	1	1	1	1	0	0	0	0	1
PB0062	1	1	1	1	1	1	1	1	1	1	1	1	0	0	0	0	0
PB0063	1	1	1	1	1	1	1	1	1	1	1	0	1	1	1	1	0
PB0064	1	1	1	1	1	1	1	1	1	1	1	0	1	0	1	0	1
PB0065	1	1	1	1	1	1	1	1	1	1	1	1	1	1	1	1	1
PB0066	1	1	1	1	1	1	1	1	1	1	1	1	1	1	0	1	1
PB0067	1	1	1	1	1	1	1	1	1	1	1	1	1	1	1	1	1
PB0068	1	1	1	1	1	1	1	1	1	1	1	0	1	0	0	0	0
PB0069	1	1	1	1	1	1	1	1	1	1	1	0	1	1	1	1	1
PB0070	1	1	1	1	1	1	1	1	1	1	1	1	1	1	1	1	1
PB0071	1	1	1	1	1	1	1	1	1	1	1	0	0	0	0	0	0
PB0072	1	1	1	1	1	1	1	1	1	1	1	1	1	1	1	1	1
PB0073	1	0	1	1	1	1	1	1	1	1	1	0	0	0	0	0	0
PB0074	1	1	1	1	1	1	1	1	1	1	1	1	1	1	1	1	1
PB0075	1	1	1	1	1	1	1	1	1	1	1	1	1	1	1	1	1
PB0076	1	1	1	1	1	1	1	1	1	1	1	1	1	1	1	1	1
PB0077	1	1	1	1	1	1	1	1	1	1	1	1	1	1	0	1	1
PB0078	1	1	1	1	1	1	1	1	1	1	1	0	0	0	0	0	1
PB0079	1	1	1	1	1	1	1	1	1	1	1	1	1	1	0	1	1
PB0080	1	1	1	1	1	1	1	1	1	1	1	1	1	1	1	1	1
PB0081	1	1	1	1	1	1	1	1	1	1	1	1	0	0	0	0	0
PB0082	1	1	1	1	1	1	1	1	1	1	1	1	1	1	1	1	1
PB0083	1	0	1	1	1	1	1	1	1	1	1	1	1	1	1	1	1
PB0084	1	1	1	1	1	1	1	1	1	1	1	1	1	1	0	1	1
PB0085	1	1	1	1	1	1	1	1	1	1	1	0	0	0	0	0	0
PB0086	1	1	1	1	1	1	1	1	1	1	1	0	0	0	0	0	0
PB0087	1	1	1	1	0	0	0	1	1	0	1	0	0	0	0	0	0

PB0088	1	1	1	1	1	1	1	0	0	1	1	0	0	0	0	0	0
PB0089	1	1	1	1	1	1	1	1	1	1	1	0	0	0	0	0	0
PB0090	1	1	1	1	1	1	1	1	1	1	1	1	0	0	0	1	0
PB0091	1	1	1	1	1	1	1	1	1	1	1	0	1	1	0	0	0
PB0092	1	1	1	1	1	1	1	1	1	1	1	1	1	1	0	0	1
PB0093	1	1	1	1	1	1	1	1	1	1	1	1	1	0	0	1	0
PB0094	1	1	1	1	1	1	1	1	1	1	1	1	0	0	0	0	0
PB0095	1	1	1	0	1	1	0	1	1	1	1	1	1	1	0	0	1
PB0096	1	1	1	1	1	1	1	1	1	1	1	1	0	0	0	0	1
PB0097	1	0	1	1	1	1	1	1	1	1	1	1	1	1	1	1	1
PB0098	1	1	1	1	1	1	1	1	1	1	1	1	1	1	1	1	1
PB0099	1	1	1	1	1	1	1	1	1	1	1	1	0	0	0	0	0
PB0100	1	1	1	1	1	1	1	1	1	1	1	1	1	1	1	1	1
PB0101	1	1	1	1	1	1	1	1	1	1	1	1	0	0	0	1	1
PB0102	1	1	1	1	1	1	1	1	1	0	1	0	0	0	0	0	0
PB0103	1	1	1	1	1	1	1	1	1	1	1	1	1	1	0	0	1
PB0104	1	1	1	1	1	1	1	1	1	1	1	1	1	1	1	1	1
PB0105	1	1	1	1	1	1	1	1	1	1	1	1	1	1	1	1	1
PB0106	1	1	1	1	1	1	1	1	1	1	1	0	0	0	0	0	0
PB0107	1	1	1	1	1	1	1	1	1	1	1	1	1	1	1	1	1
PB0108	1	1	1	1	1	1	1	1	1	1	1	1	1	1	0	1	1
PB0109	1	1	1	1	1	1	1	1	1	1	1	0	0	0	0	0	0
PB0110	1	1	1	1	1	1	1	1	1	1	1	1	1	1	1	1	1
PB0111	1	1	1	1	1	1	1	1	1	1	1	1	1	1	0	1	1
PB0112	1	1	1	1	1	1	1	1	1	1	1	1	1	1	1	1	1
PB0113	1	1	1	1	1	1	1	1	1	1	1	1	1	1	1	1	1
PB0114	1	1	1	1	1	1	1	1	1	1	1	1	0	0	0	0	1
PB0115	1	1	1	1	1	1	1	1	1	1	1	0	0	0	0	0	0
PB0116	1	1	1	1	1	1	1	1	1	1	1	1	1	1	1	1	1
PB0117	1	1	1	1	1	1	1	1	1	1	1	1	1	1	1	1	1
PB0118	1	1	1	1	1	1	1	1	1	1	1	1	1	0	0	0	1
PB0119	1	1	1	1	1	1	1	1	1	1	1	1	1	1	1	1	1
PB0120	1	1	1	1	1	1	1	1	1	1	1	1	0	0	0	0	0
PB0121	1	1	1	1	1	1	1	1	1	1	1	1	1	0	0	0	0
PB0122	1	1	1	1	1	1	1	1	1	1	1	1	1	1	0	1	1
PB0123	1	1	1	1	1	1	1	1	1	1	1	1	1	1	1	0	1
PB0124	1	1	1	1	1	1	1	1	1	1	1	1	1	1	1	1	1
PB0125	1	1	1	1	1	1	1	1	1	1	1	1	1	1	1	0	1
PB0126	1	1	0	0	0	0	0	1	1	1	1	0	0	0	0	0	0
PB0127	1	1	1	1	1	1	1	1	1	1	1	0	1	1	0	0	0
PB0128	1	1	1	1	1	1	1	1	1	1	1	1	1	1	1	1	1
PB0129	1	1	1	1	1	1	1	1	1	1	1	1	1	1	1	1	1
PB0130	1	1	1	1	1	1	1	1	1	1	1	0	1	1	1	0	0
PB0131	1	1	1	1	1	1	1	1	1	1	1	1	1	1	1	0	0
PB0132	1	1	1	1	1	1	1	1	1	1	1	1	1	1	1	0	1
PB0133	1	0	1	1	1	1	1	1	1	1	1	0	0	0	0	0	0
PB0134	1	1	1	1	1	1	1	1	1	1	1	1	0	0	0	1	1
PB0135	1	1	1	1	1	1	1	1	1	1	1	1	1	1	1	0	1
PB0136	1	1	1	1	1	1	1	1	1	1	1	1	1	1	0	0	1
PB0137	1	1	1	1	1	1	1	1	1	1	1	1	1	1	1	1	1
PB0138	1	1	1	1	1	1	1	1	1	1	1	1	1	1	1	1	0
PB0139	1	1	1	1	1	1	1	1	1	1	1	0	0	1	0	0	1
PB0140	1	1	1	1	1	1	1	1	1	1	1	1	1	1	1	1	1
PB0141	1	1	1	1	1	1	1	1	1	1	1	1	1	1	1	0	0
PB0142	1	1	1	1	1	1	1	1	1	1	1	1	1	1	1	1	0
PB0143	1	1	1	1	1	1	1	1	1	1	1	1	1	1	1	0	0
PB0144	1	1	1	1	1	1	1	1	1	1	1	0	1	1	0	1	1
PB0145	1	0	1	1	1	1	1	1	1	1	1	0	1	1	1	1	1

PB0146	1	1	1	1	1	1	1	1	1	1	1	1	1	1	0	1	1
PB0147	1	1	1	1	1	1	1	1	1	1	1	1	1	1	1	1	1
PB0148	1	1	1	1	1	1	1	1	1	1	1	1	1	1	1	0	1
PB0149	1	1	1	1	1	1	1	1	1	1	1	1	1	1	1	1	1
PB0150	1	1	1	1	1	1	1	1	1	1	1	1	1	1	1	0	1
PB0151	1	1	1	1	1	1	1	1	1	1	1	1	1	1	0	1	1
PB0152	1	1	1	1	1	1	1	1	1	1	1	1	1	1	1	0	1
PB0153	1	1	1	1	1	1	1	1	1	1	1	1	1	1	1	1	1
PB0154	1	1	1	1	1	1	1	1	1	1	1	0	0	1	0	0	0
PB0155	1	1	1	1	1	1	1	1	1	1	1	1	0	1	1	0	1
PB0156	1	1	1	1	1	1	1	1	1	1	1	1	1	1	0	1	1
PB0157	1	1	1	1	1	1	1	1	1	1	1	1	0	0	0	0	0
PB0158	1	1	1	1	1	1	1	1	1	1	1	1	0	0	0	0	0
PB0159	1	1	1	1	1	1	1	1	1	1	1	1	1	0	0	0	1
PB0160	1	1	1	1	1	1	1	1	1	1	1	1	1	0	0	0	1
PB0161	1	1	1	1	1	1	1	1	1	1	1	1	1	1	1	1	1
PB0162	1	1	1	1	1	1	1	1	1	1	1	1	1	0	0	0	0
PB0163	1	1	1	1	1	1	1	1	1	1	1	1	0	0	0	0	0
PB0164	1	1	1	1	1	1	1	1	1	1	1	1	1	1	0	1	1
PB0165	1	1	1	1	1	1	1	1	1	1	1	1	1	1	1	0	1
PB0166	1	1	1	1	1	1	1	1	1	1	1	1	1	0	0	0	0
PB0167	1	0	1	1	1	1	1	1	1	1	1	1	1	1	1	1	1
PB0168	1	1	1	1	1	1	1	1	1	1	1	1	1	1	1	0	0
PB0169	1	1	1	1	1	1	1	1	1	1	1	1	1	1	0	0	1
PB0170	1	1	1	1	1	1	1	1	1	1	1	1	1	1	0	1	1
PB0171	1	1	1	1	1	1	1	1	1	1	1	1	1	1	0	1	0
PB0172	1	1	1	1	1	1	1	1	1	1	1	0	0	0	0	0	0
PB0173	1	0	1	1	1	1	1	1	1	1	1	1	1	1	1	1	1
PB0174	1	1	1	1	1	1	1	1	1	1	1	1	1	1	0	0	0
PB0175	1	1	1	1	1	1	1	1	1	1	1	1	1	1	1	0	1
PB0176	1	1	1	1	1	1	1	1	1	1	1	0	0	1	0	1	1
PB0177	1	1	1	1	1	1	1	1	1	1	1	1	0	0	0	0	1
PB0178	1	1	1	1	1	1	1	1	1	1	1	1	1	1	1	1	1
PB0179	1	1	1	1	1	1	1	1	1	1	1	0	0	0	0	0	0
PB0180	1	1	1	1	1	1	1	1	1	1	1	1	0	0	0	0	1
PB0181	1	1	1	1	1	1	1	1	1	1	1	1	0	0	0	0	0
PB0182	1	1	1	1	1	1	1	1	1	1	1	1	1	0	0	0	0
PB0183	1	0	1	1	1	1	1	1	1	1	1	1	1	1	1	1	0
PB0184	1	1	1	1	1	1	1	1	1	1	1	1	1	1	1	0	1
PB0185	1	1	1	1	1	1	1	1	1	1	1	1	1	1	0	0	0
PB0186	1	1	1	1	1	1	1	1	1	1	1	1	1	1	1	1	1
PB0187	1	1	1	1	1	1	1	1	1	1	1	0	0	0	0	0	0
PB0188	1	1	1	1	1	1	1	1	1	1	1	1	1	1	0	0	1
PB0189	1	1	1	1	1	1	1	1	1	1	1	0	0	1	0	0	1
PB0190	1	1	1	1	1	1	1	1	1	1	1	1	1	0	0	0	0
PB0191	1	1	1	1	1	1	1	1	1	1	1	0	0	0	0	0	0
PB0192	1	1	1	1	1	1	1	1	1	1	1	1	1	1	0	0	1
PB0193	1	1	1	1	1	1	1	1	1	1	1	1	1	1	1	0	0
PB0194	1	1	1	1	1	1	1	1	1	1	1	1	0	1	0	0	0
PB0195	1	1	1	1	1	1	1	1	1	1	1	1	1	1	0	0	1
PB0196	1	1	1	1	1	1	1	1	1	1	1	1	0	1	0	0	0
PB0197	1	1	1	1	1	1	1	1	1	1	1	1	1	1	0	0	1
PB0198	1	1	1	1	1	1	1	1	1	1	1	1	1	1	1	0	1
PB0199	1	0	1	0	1	1	0	1	1	1	1	1	1	1	1	0	1
PB0200	1	1	1	1	1	1	1	1	1	1	1	0	0	0	0	0	0
PB0201	1	1	1	1	1	1	1	1	1	1	1	1	1	1	1	0	1
PB0202	1	1	1	1	1	1	1	1	1	1	1	1	1	0	0	0	1
PB0203	1	1	1	1	1	1	1	0	1	1	1	1	1	1	1	0	1

PB0204	1	1	1	1	1	1	1	1	1	1	1	1	1	1	0	1	1
PB0205	1	1	1	1	1	1	1	1	1	1	1	1	1	1	1	0	1
PB0206	1	1	1	1	1	1	1	1	1	1	1	0	0	0	0	0	0
PB0207	1	1	1	1	1	1	1	1	1	1	1	0	1	0	1	1	1
PB0208	1	1	1	1	1	1	1	1	1	1	1	1	1	1	1	1	1
PB0209	1	1	1	1	1	1	1	1	1	1	1	1	1	1	1	1	1
PB0210	1	1	1	1	1	1	1	1	1	0	0	0	0	0	0	0	0
PB0211	1	1	1	1	1	1	1	1	1	1	1	1	1	1	1	1	1
PB0212	1	1	1	1	1	1	1	1	1	1	1	0	0	0	0	0	0
PB0213	1	1	1	1	1	1	1	1	1	1	1	1	1	0	1	1	1
PB0214	1	1	1	1	1	1	1	1	1	1	1	1	1	1	1	1	1
PB0215	1	1	1	1	1	1	1	1	1	1	1	1	1	1	1	0	1
PB0216	1	1	1	1	1	1	1	1	1	1	1	0	1	1	0	1	0
PB0217	1	0	1	1	1	1	1	1	1	1	1	0	0	0	0	0	0
PB0218	1	1	1	1	1	1	1	1	1	1	1	0	0	0	0	0	0
PB0219	1	1	1	1	1	1	1	1	1	1	1	1	0	0	0	0	0
PB0220	1	1	1	1	1	1	1	1	1	1	1	1	1	0	0	0	1
PB0221	1	1	1	1	1	1	1	1	1	1	1	0	1	1	0	1	0
PB0222	1	1	1	1	1	1	1	1	1	1	1	0	0	1	0	0	0
PB0223	1	1	1	1	1	1	1	1	1	1	1	1	1	1	1	0	1
PB0224	1	1	1	1	1	1	1	1	1	1	1	1	0	1	0	0	0
PB0225	1	1	1	1	1	1	1	1	1	1	1	1	1	1	0	1	1
PB0226	1	1	1	1	1	1	1	1	1	1	1	1	1	1	0	1	1
PB0227	1	1	1	1	1	1	1	1	1	1	1	1	1	1	0	1	0
PB0228	1	1	1	1	1	1	1	1	1	1	1	0	0	0	0	0	0
PB0229	1	0	1	1	1	1	1	1	1	1	1	0	0	1	0	0	0
PB0230	1	1	1	1	1	1	1	1	1	1	1	0	0	1	0	0	1
PB0231	1	1	1	1	1	1	1	1	1	1	1	1	1	1	1	1	1
PB0232	1	1	1	1	1	1	1	1	1	1	1	1	0	0	0	0	1
PB0233	1	1	1	1	1	1	1	1	1	1	1	1	0	1	0	0	1
PB0234	1	1	1	1	1	1	1	1	1	1	1	1	1	1	1	1	1
PB0235	1	1	1	1	1	1	1	1	1	1	1	1	1	1	1	1	1
PB0236	1	1	1	1	1	1	1	1	1	1	1	1	1	1	1	1	1
PB0237	1	1	1	1	1	1	1	1	1	1	1	1	1	1	1	1	1
PB0238	1	1	1	1	1	1	1	1	1	1	1	1	0	0	0	0	0
PB0239	1	1	1	1	1	1	1	1	1	1	1	1	1	1	1	1	1
PB0240	1	1	1	1	1	1	1	1	1	1	1	1	1	1	1	0	1
PB0241	1	1	1	1	1	1	1	1	1	1	1	1	1	0	0	1	1
PB0242	1	1	1	1	1	1	1	1	1	1	1	0	0	0	0	0	0
PB0243	1	1	1	1	1	1	1	1	1	1	1	0	1	0	0	0	0
PB0244	1	1	1	1	1	1	1	1	1	1	1	0	1	1	1	0	0
PB0245	1	1	1	1	1	1	1	1	1	1	1	1	1	0	1	0	1
PB0246	1	1	1	1	1	1	1	1	1	1	1	1	1	1	1	1	1
PB0247	1	1	1	1	1	1	1	1	1	1	1	1	1	1	0	0	1
PB0248	1	1	1	1	1	1	1	1	1	1	1	1	1	1	1	1	1
PB0249	1	1	1	1	1	1	1	1	1	0	0	0	0	0	0	0	0
PB0250	1	1	1	1	1	1	1	1	1	1	0	0	0	0	0	0	0
PB0251	1	1	1	1	1	1	1	1	1	1	1	1	1	1	0	0	1
PB0252	1	1	1	1	1	1	1	1	1	1	1	1	1	0	0	1	0
PB0253	1	1	1	1	1	1	1	1	1	1	1	1	1	0	0	1	0
PB0254	1	1	1	1	1	1	1	1	1	1	1	1	1	0	0	1	0
PB0255	1	1	1	1	1	1	1	1	1	1	1	1	1	0	0	1	0

N: Number of species; Pm: Primate; Ra: Rabbit; Ro: Rodent; Ca: Carnivore; De: Deer; Bo: Bovid; Pi: Pig; Re: Reptile; Bi: Bird; Am: amphibian; Fi: Bony fishes; Tu: Tunicates; Ec: Echinoderm; In: Insects; Ne: Nematode; Ce: Cephalopod; and Cn: Cnidarian

Supplementary table S4. Domain present in human bone extracellular matrix proteins dataset. Data of domains in other species is available from the database.

Domain	N	Type	Description
PF01391_Collagen	30	Repeat	Collagen triple helix repeat
PF13855_LRR_8	16	Repeat	Leucine rich repeat
PF00089_Trypsin	15	Domain	Trypsin
PF00092_VWA	15	Domain	von Willebrand factor type A domain
PF00008_EGF	14	Domain	EGF-like domain
PF07645_EGF_CA	13	Domain	Calcium-binding EGF domain
PF00053_Laminin_EGF	10	Domain	Laminin EGF domain
PF00093_VWC	9	Domain	von Willebrand factor type C domain
PF12662_cEGF	9	Domain	Complement Clr-like EGF-like
PF00594_Gla	8	Domain	Vitamin K-dependent carboxylation/gamma-carboxyglutamic (GLA) domain
PF01410_COLFI	8	Family	Fibrillar collagen C-terminal domain
PF01462_LRRNT	8	Family	Leucine rich repeat N-terminal domain
PF02210_Laminin_G_2	8	Domain	Laminin G domain
PF00041_fn3	7	Domain	Fibronectin type III domain
PF00079_Serpin	7	Domain	Serpin (serine protease inhibitor)
PF00147_Fibrinogen_C	7	Domain	Fibrinogen beta and gamma chains, C-terminal globular domain
PF01759_NTR	7	Domain	UNC-6/NTR/C345C module
PF00054_Laminin_G_1	6	Domain	Laminin G domain
PF00055_Laminin_N	6	Domain	Laminin N-terminal (Domain VI)
PF00084_Sushi	6	Domain	Sushi repeat (SCR repeat)
PF00090_TSP_1	6	Domain	Thrombospondin type 1 domain
PF00219_IGFBP	6	Domain	Insulin-like growth factor binding protein
PF00019_TGF_beta	5	Domain	Transforming growth factor beta like domain
PF00094_VWD	5	Domain	von Willebrand factor type D domain
PF01392_Fz	5	Domain	Fz domain
PF01413_C4	5	Domain	C-terminal tandem repeated domain in type 4 procollagen
PF07648_Kazal_2	5	Domain	Kazal-type serine protease inhibitor domain
PF00045_Hemopexin	4	Repeat	Hemopexin
PF00052_Laminin_B	4	Domain	Laminin B (Domain IV)
PF00059_Lectin_C	4	Domain	Lectin C-type domain
PF00086_Thyroglobulin_1	4	Domain	Thyroglobulin type-1 repeat
PF00193_Xlink	4	Domain	Extracellular link domain
PF00688_TGFb_propeptide	4	Family	TGF-beta propeptide
PF01826_TIL	4	Domain	Trypsin Inhibitor like cysteine rich domain
PF02412_TSP_3	4	Domain	Thrombospondin type 3 repeat
PF05735_TSP_C	4	Family	Thrombospondin C-terminal region
PF08742_C8	4	Domain	C8 domain
PF15913_Furin-like_2	4	Domain	Furin-like repeat, cysteine-rich
PF00014_Kunitz_BPTI	3	Domain	Kunitz/Bovine pancreatic trypsin inhibitor domain
PF00031_Cystatin	3	Domain	Cystatin domain
PF00040_fn2	3	Domain	Fibronectin type II domain
PF00051_Kringle	3	Domain	Kringle domain
PF00191_Annexin	3	Domain	Annexind
PF00207_A2M	3	Family	Alpha-2-macroglobulin family
PF00307_CH	3	Domain	Calponin homology (CH) domain
PF00386_C1q	3	Domain	C1q domain
PF00413_Peptidase_M10	3	Domain	Matrixin
PF00431_CUB	3	Domain	CUB domain
PF00683_TB	3	Family	TB domain
PF01442_Apolipoprotein	3	Domain	Apolipoprotein A1/A4/E domain
PF01471_PG_binding_1	3	Domain	Putative peptidoglycan binding domain
PF01835_MG2	3	Domain	MG2 domain
PF02191_OLF	3	Repeat	Olfactomedin-like domain

PF06008_Laminin_I	3	Coiled-coil	Laminin Domain I
PF06009_Laminin_II	3	Coiled-coil	Laminin Domain II
PF07677_A2M_recep	3	Domain	A-macroglobulin receptor binding domain
PF07678_TED_complement	3	Repeat	A-macroglobulin TED domain
PF07686_V-set	3	Domain	Immunoglobulin V-set domain
PF07703_A2M_BRD	3	Domain	Alpha-2-macroglobulin bait region domain
PF07974_EGF_2	3	Domain	EGF-like domain
PF08702_Fib_alpha	3	Coiled-coil	Fibrinogen alpha/beta chain family
PF09289_FOLN	3	Domain	Follistatin/Osteonectin-like EGF domain
PF10393_Matrilin_ccoil	3	Coiled-coil	Trimeric coiled-coil oligomerisation domain of matrilin
PF12661_hEGF	3	Domain	Human growth factor-like EGF
PF12947_EGF_3	3	Domain	EGF domain
PF13330_Mucin2_WxxW	3	Family	Mucin-2 protein WxxW repeating region
PF13833_EF-hand_8	3	Domain	EF-hand domain pair
PF17789_MG4	3	Domain	Macroglobulin domain MG4
PF17791_MG3	3	Domain	Macroglobulin domain MG3
PF18720_EGF_Tenascin	3	Domain	Tenascin EGF domain
PF06119_NIDO	3	Family	Nidogen-like
PF00050_Kazal_1	2	Domain	Kazal-type serine protease inhibitor domain
PF00057_Ldl_recept_a	2	Repeat	Low-density lipoprotein receptor domain class A
PF00058_Ldl_recept_b	2	Repeat	Low-density lipoprotein receptor repeat class B
PF00061_Lipocalin	2	Domain	Lipocalin / cytosolic fatty-acid binding protein family
PF00106_adh_short	2	Domain	Short chain dehydrogenase
PF00405_Transferrin	2	Domain	Transferrin
PF00530_SRCR	2	Domain	Scavenger receptor cysteine-rich domain
PF00560_LRR_1	2	Repeat	Leucine Rich Repeat
PF00626_Gelsolin	2	Domain	Gelsolin repeat
PF00648_Peptidase_C2	2	Family	Calpain family cysteine protease
PF01033_Somatomedin_B	2	Family	Somatomedin B domain
PF01067_Calpain_III	2	Domain	Calpain large subunit, domain III
PF01186_Lysyl_oxidase	2	Family	Lysyl oxidase
PF01463_LRRCT	2	Family	Leucine rich repeat C-terminal domain
PF01821_ANATO	2	Domain	Anaphylotoxin-like domain
PF01839_FG-GAP	2	Repeat	FG-GAP repeat
PF02469_Fasciclin	2	Domain	Fasciclin domain
PF04089_BRICHOS	2	Domain	BRICHOS domain
PF07474_G2F	2	Domain	G2F domain
PF08441_Integrin_alpha2	2	Family	Integrin alpha
PF11598_COMP	2	Family	Cartilage oligomeric matrix protein
PF12714_TILa	2	Domain	TILa domain
PF13499_EF-hand_7	2	Domain	EF-hand domain pair
PF13516_LRR_6	2	Repeat	Leucine Rich repeat
PF13778_DUF4174	2	Family	Domain of unknown function (DUF4174)
PF17790_MG1	2	Domain	Macroglobulin domain MG1
PF17820_PDZ_6	2	Domain	PDZ domain
PF18193_Fibrillin_U_N	2	Domain	Fibrillin 1 unique N-terminal domain
PF00754_F5_F8_type_C	2	Domain	F5/8 type C domain
PF00246_Peptidase_M14	2	Domain	Zinc carboxypeptidase
PF19028_TSP1_spondin	2	Domain	Spondin-like TSP1 domain
PF00007_Cys_knot	1	Domain	Cystine-knot domain
PF00012_HSP70	1	Family	Hsp70 protein
PF00018_SH3_1	1	Domain	SH3 domain
PF00024_PAN_1	1	Domain	PAN domain
PF00039_fn1	1	Domain	Fibronectin type I domain
PF00042_Globin	1	Domain	Globin
PF00044_Gp_dh_N	1	Domain	Glyceraldehyde 3-phosphate dehydrogenase, NAD binding domain
PF00048_IL8	1	Domain	Small cytokines (intecrine/chemokine), interleukin-8 like
PF00062_Lys	1	Domain	C-type lysozyme/alpha-lactalbumin family

PF00071_Ras	1	Domain	Ras family
PF00080_Sod_Cu	1	Domain	Copper/zinc superoxide dismutase (SODC)
PF00085_Thioredoxin	1	Domain	Thioredoxin
PF00100_Zona_pellucida	1	Family	Zona pellucida-like domain
PF00112_Peptidase_C1	1	Domain	Papain family cysteine protease
PF00167_FGF	1	Domain	Fibroblast growth factor
PF00169_PH	1	Domain	PH domain
PF00210_Ferritin	1	Domain	Ferritin-like domain
PF00245_Alk_phosphatase	1	Domain	Alkaline phosphatase
PF00354_Pentaxin	1	Domain	Pentaxin family
PF00357_Integrin_alpha	1	Family	Integrin alpha cytoplasmic region
PF00362_Integrin_beta	1	Domain	Integrin beta chain VWA domain
PF00373_FERM_M	1	Domain	FERM central domain
PF00412_LIM	1	Domain	LIM domain
PF00435_Spectrin	1	Domain	Spectrin repeat
PF00445_Ribonuclease_T2	1	Domain	Ribonuclease T2 family
PF00505_HMG_box	1	Domain	HMG (high mobility group) box
PF00514_Arm	1	Repeat	Armadillo
PF00576_Transthyretin	1	Domain	HIUase/Transthyretin family
PF00578_AhpC-TSA	1	Domain	AhpC/TSA family
PF00594_GLA	1	Domain	Vitamin K-dependent carboxylation/gamma-carboxyglutamic (GLA) domain
PF00630_Filamin	1	Domain	Filamin/ABP280 repeat
PF00787_PX	1	Domain	PX domain
PF00865_Osteopontin	1	Family	Osteopontin
PF00965_TIMP	1	Domain	Tissue inhibitor of metalloproteinase
PF01023_S_100	1	Domain	S-100/ICaBP type calcium binding domain
PF01044_Vinculin	1	Family	Vinculin family
PF01091_PTN_MK_C	1	Domain	PTN/MK heparin-binding protein family, C-terminal domain
PF01093_Clusterin	1	Family	Clusterin
PF01122_Cobalamin_bind	1	Repeat	Eukaryotic cobalamin-binding protein
PF01223_Endonuclease_NS	1	Domain	DNA/RNA non-specific endonuclease
PF01347_Vitellogenin_N	1	Repeat	Lipoprotein amino terminal region
PF01390_SEA	1	Family	SEA domain
PF01400_Astacin	1	Domain	Astacin (Peptidase family M12A)
PF01549_ShK	1	Domain	ShK domain-like
PF01663_Phosphodiest	1	Family	Type I phosphodiesterase / nucleotide pyrophosphatase
PF01731_Arylesterase	1	Repeat	Arylesterase
PF02014_Reeler	1	Family	Reeler domain
PF02177_APP_N	1	Domain	Amyloid A4 N-terminal heparin-binding
PF02494_HYR	1	Domain	HYR domain
PF02800_Gp_dh_C	1	Domain	lyceraldehyde 3-phosphate dehydrogenase, C-terminal domain
PF03045_DAN	1	Domain	DAN domain
PF03146_NtA	1	Domain	Agrin NtA domain
PF03494_Beta-APP	1	Family	Beta-amyloid peptide (beta-APP)
PF03815_LCCL	1	Domain	LCCL domain
PF04587_ADP_PFK_GK	1	Family	ADP-specific Phosphofructokinase/Glucokinase conserved region
PF04691_ApoC-I	1	Family	Apolipoprotein C-I (ApoC-1)
PF04706_Dickkopf_N	1	Family	Dickkopf N-terminal cysteine-rich region
PF04711_ApoA-II	1	Family	Apolipoprotein A-II (ApoA-II)
PF04777_Evr1_Alr	1	Family	Erv1 / Alr family
PF05196_PTN_MK_N	1	Domain	PTN/MK heparin-binding protein family, N-terminal domain
PF05355_Apo-CII	1	Family	Apolipoprotein C-II
PF05432_BSP_II	1	Family	Bone sialoprotein II (BSP-II)
PF05507_MAGP	1	Family	Microfibril-associated glycoprotein (MAGP)
PF06121_DUF959	1	Domain	Domain of Unknown Function (DUF959)
PF06448_DUF1081	1	Family	Domain of Unknown Function (DUF1081)
PF06468_Spond_N	1	Domain	Spondin_N
PF06473_FGF-BP1	1	Family	FGF binding protein 1 (FGF-BP1)

PF06482_Endostatin	1	Domain	Collagenase NC10 and Endostatin
PF06668_ITI_HC_C	1	Family	Inter-alpha-trypsin inhibitor heavy chain C-terminus
PF06951_PLA2G12	1	Domain	Group XII secretory phospholipase A2 precursor (PLA2G12)
PF07448_Spp-24	1	Domain	Secreted phosphoprotein 24 (Spp-24) cystatin-like domain
PF07546_EMI	1	Domain	EMI domain
PF07653_SH3_2	1	Domain	Variant SH3 domain
PF07654_C1-set	1	Domain	Immunoglobulin C1-set domain
PF07679_I-set	1	Domain	Immunoglobulin I-set domain
PF07965_Integrin_B_tail	1	Domain	Integrin beta tail domain
PF08246_Inhibitor_I29	1	Domain	Cathepsin propeptide inhibitor domain (I29)
PF08391_Ly49	1	Family	Ly49-like protein, N-terminal region
PF08487_VIT	1	Family	Vault protein inter-alpha-trypsin domain
PF08725_Integrin_b_cyt	1	Domain	Integrin beta cytoplasmic domain
PF08726_EFhand_Ca_insen	1	Domain	Ca2+ insensitive EF hand
PF09011_HMG_box_2	1	Domain	HMG-box domain
PF09172_Vit_open_b-sht	1	Domain	Vitellinogen, open beta-sheet
PF09396_Thrombin_light	1	Domain	Thrombin light chain
PF10179_NDNF	1	Domain	Neuron-derived neurotrophic factor, first Fn(III) domain
PF10417_1-cysPrx_C	1	Domain	C-terminal domain of 1-Cys peroxiredoxin
PF10515_APP_amyloid	1	Family	Beta-amyloid precursor protein C-terminus
PF10591_SPARC_Ca_bdg	1	Domain	Secreted protein acidic and rich in cysteine Ca binding region
PF11032_ApoM	1	Domain	ApoM domain
PF12160_Fibrinogen_aC	1	Domain	Fibrinogen alpha C domain
PF12491_ApoB100_C	1	Family	Apolipoprotein B100 C terminal
PF12924_APP_Cu_bd	1	Domain	Copper-binding of amyloid precursor, CuBD
PF12925_APP_E2	1	Domain	E2 domain of amyloid precursor protein
PF13202_EF-hand_5	1	Domain	EF hand
PF13306_LRR_5	1	Repeat	BspA type Leucine rich repeat region (6 copies)
PF13895_Ig_2	1	Domain	Immunoglobulin domain
PF13927_IG_3	1	Domain	Immunoglobulin domain
PF14478_DUF4430	1	Domain	Domain of unknown function (DUF4430)
PF14704_DERM	1	Family	Dermatopontin
PF15182_OTOS	1	Family	Otospiralin
PF15467_SGIII	1	Family	Secretogranin-3
PF15511_CENP-T_C	1	Domain	Centromere kinetochore component CENP-T histone fold
PF15819_Fibin	1	Family	Fin bud initiation factor homologue
PF17085_UCMA	1	Family	Unique cartilage matrix associated protein
PF17205_PSI_integrin	1	Domain	Integrin plexin domain
PF17517_IgGFc_binding	1	Family	IgGFc binding protein
PF18108_QSOX_Trx1	1	Domain	QSOX Trx-like domain
PF18124_Kindlin_2_N	1	Domain	Kindlin-2 N-terminal domain
PF18371_FAD_SOX	1	Domain	Flavin adenine dinucleotide (FAD)-dependent sulfhydryl oxidase
PF18372_I-EGF_1	1	Domain	Integrin beta epidermal growth factor like domain 1
PF19035_TSP1_CCN	1	Domain	CCN3 Nov like TSP1 domain
PF20010_Collagen_trimer	1	Domain	Collagen trimerization domain

N = Number of proteins.

Supplementary table 5. List of highly conserved proteins between *Danio rerio* (zebrafish) and *Homo sapiens* (humans).

Phylobone ID	Unirpot *D. rerio*	Uniprot *H. sapiens*	Description
PB0188	Q1RLX2 (timp2a)	P16035 (TIMP2)	Complexes with metalloproteinases (such as collagenases) and irreversibly inactivates them by binding to their catalytic zinc cofactor [7]. Metalloproteinase inhibitors are able to block cartilage destruction in human disease [8]. Tissue inhibitor of matrix metalloproteinase-2 (TIMP2) genes changed reversely in deer physiological and human age-related osteoporosis [9].
PB0150	F1R2Z9 (spon1b)	Q9HCB6 (SPON1)	Major factor for vascular smooth muscle cells [7]. R-Spondin 1 (Rspo1) is able to promote osteoblastic differentiation and bone formation, protect against inflammatory bone damage and attenuate age-related bone loss. Rspo1 as a mechanosensitive protein in the upstream of the Wnt/β-catenin signaling pathway and highlighted the potential role of the Rspo1 and Rspo1/Lgr4 signal in bone mechanotransduction [6]. Rspo 1 promotes bone formation in three mouse models of osteoporosis [10].
PB0192	Q2PMI2 (fstl1b)	Q12841 (FSTL1)	Initiates various signaling cascades by activating different receptors on the cell surface such as DIP2A, TLR4 or BMP receptors [7]. Follistatin-like protein 1 (FSTL1) is a secreted glycoprotein that has been implicated in arthritis pathogenesis in a mouse model. FSTL1 is involved in various pathological and physiological processes, such as growth factors, immune modulation, and cell proliferation and differentiation [11]. FSTL1 is an osteogenic suppressor that inhibits the osteogenic differentiation of mesenchymal stem cells (MSCs) during inflammation and it can be a new target for bone regeneration [12].
PB0059	B8JLZ3 (anxa1b)	P04083 (ANXA1)	Plays a role in the formation of phagocytic cups and phagosomes. Plays a role in phagocytosis by mediating the Ca2+-dependent interaction between phagosomes and the actin cytoskeleton (By similarity) [7]. Annexin A1 (ANXA1) is involved in regulating bone development and the bone marrow microenvironment [13].
PB0201	Q66I23 (tgfb3)	P10600 (TGFB3)	Transforming growth factor beta-3 (TGF-beta-3) proprotein: Precursor of the Latency-associated peptide (LAP) and TGF-beta-3 chains, which constitute the regulatory and active subunit of TGF-beta-3, respectively. [7]. As the bone matrix deposited by osteoblasts contains abundant TGF-β in its latent form (~200 µg/kg), the acidic environment created by osteoclasts offers an ideal condition for TGF-β activation [14]. TGF-beta-3 is a growth factor involved in bone generation and its overexpression upregulates alkaline phosphatase activity and induces the osteogenic differentiation of MSCs. It also induces chondrogenesis of MSCs [15].
PB0036	A9JRB3 (htra1b)	Q92743 (HTRA1)	Serine protease with a variety of targets, including extracellular matrix proteins such as fibronectin. [7] High-temperature requirement serine protease A1 (HTRA1) is a secreted member of the trypsin family of serine proteases which can degrade a variety of bone matrix proteins and as such has been implicated in musculoskeletal development. Recombinant HTRA1 enhanced the osteogenesis of MSCs as evidenced by significant changes in several osteogenic markers including integrin-binding sialoprotein (IBSP), bone morphogenetic protein 5 (BMP5), and sclerostin, and promoted matrix mineralization in differentiating bone-forming osteoblasts [16].
PB0196	Q5RI33 (ctgfa)	P29279 (CTGF)	Major connective tissue mitoattractant secreted by vascular endothelial cells. CTGF promotes proliferation and differentiation of chondrocytes. CTGF mediates heparin- and divalent cation-dependent cell adhesion in many cell types including fibroblasts, myofibroblasts, endothelial and epithelial cells. [7]. CTGF plays a critical role in intramembranous osteogenesis through induction of proliferation and differentiation of osteoblast lineage cells, secretion and mineralization of bone matrix and chemoattractant for osteoblast lineage cells in intramembranous osteogenesis [17].
PB0006	Q6PVV8 (sparc)	P09486 (SPARC)	Appears to regulate cell growth through interactions with the extracellular matrix and cytokines. Binds calcium and copper, several types of collagen, albumin, thrombospondin, PDGF and cell membranes [7]. Secreted protein acidic and rich in cysteine (SPARC) is a glycoprotein in the bone matrix, also known as osteonectin. SPARC is present in mineralized tissues and highly expressed in osteoblasts of bone [18]. In the osteoid, SPARC binds to collagen and hydroxyapatite crystals and releases calcium ions to enhance mineralization of the collagen matrix in bones [19].
PB0130	F1QPX0 (ndnf)	Q8TB73 (NDNF)	Promotes endothelial cell survival, vessel formation and plays an important role in the process of revascularization through NOS3-dependent mechanisms [7]. NDNF deficiency exacerbates skeletal muscle atrophy of mice induced by dexamethasone treatment or sciatic denervation [20].
PB0173	F1REM3 (bmp1a)	P13497 (BMP1)	Additional substrates include matricellular thrombospondin-1/THBS1 whose cleavage leads to cell adhesion disruption and TGF-beta activation [7]. Bone Morphogenetic protein 1 (BMP1) differs from all other BMPs, in that it is not a TGFβ-like protein. Rather, it contains a metalloproteinase domain [21]. BMP1 promotes osteogenic differentiation of MSCs [22,23]

Supplementary table 6. List of proteins that are targets of existing drugs in the market.

Drugs \ Uniprot codes	O14793	P00734	P00740	P00742	P00747	P00751	P01009	P01024	P01031	P01042	P02743	P02753	P02776	P04114	P05121	P05186	P05556	P06756	P07477	P08253	P08648	P10600	P10909	P14780	P21333	P29279	P43235	P45452	P61812	P63000	P69905	Q92743	Q9BX93	Q9BXY4	Q9GZV9	Q9UNI1
D00160 - Aminocaproic acid (USP/INN); Epsilon-Aminocaproic acid (JAN); Amicar (TN)	0	0	0	0	1	0	0	0	0	0	0	0	0	0	0	0	0	0	0	0	0	0	0	0	0	0	0	0	0	0	0	0	0	0	0	0
D00181 - Argatroban (USP); Argatroban hydrate (JP18); Novastan (TN)	0	1	0	0	0	0	0	0	0	0	0	0	0	0	0	0	0	0	0	0	0	0	0	0	0	0	0	0	0	0	0	0	0	0	0	0
D01136 - Tranexamic acid (JP18/USP/INN); Cyklokapron (TN); Rikavarin (TN); Transamin (TN)	0	0	0	0	1	0	0	0	0	0	0	0	0	0	0	0	0	0	0	0	0	0	0	0	0	0	0	0	0	0	0	0	0	0	0	0
D01844 - Fondaparinux sodium (JAN/USP/INN); Arixtra (TN)	0	0	0	1	0	0	0	0	0	0	0	0	0	0	0	0	0	0	0	0	0	0	0	0	0	0	0	0	0	0	0	0	0	0	0	0
D01981 - Ximelagatran (JAN/USAN/INN); Exanta (TN)	0	1	0	0	0	0	0	0	0	0	0	0	0	0	0	0	0	0	0	0	0	0	0	0	0	0	0	0	0	0	0	0	0	0	0	0
D03061 - Batimastat (USAN/INN)	0	0	0	0	0	0	0	0	0	0	0	0	0	0	0	0	0	0	0	1	0	0	0	1	0	0	0	0	0	0	0	0	0	0	0	0
D03136 - Bivalirudin (USAN/INN)	0	1	0	0	0	0	0	0	0	0	0	0	0	0	0	0	0	0	0	0	0	0	0	0	0	0	0	0	0	0	0	0	0	0	0	0
D03213 - Apixaban (JAN/USAN/INN); Eliquis (TN)	0	0	0	1	0	0	0	0	0	0	0	0	0	0	0	0	0	0	0	0	0	0	0	0	0	0	0	0	0	0	0	0	0	0	0	0
D03330 - Kallidinogenase (JP18/INN); Cacelmin A (TN)	0	0	0	0	0	0	0	0	0	1	0	0	0	0	0	0	0	0	0	0	0	0	0	0	0	0	0	0	0	0	0	0	0	0	0	0
D03436 - Cellulose; oxidized (JAN/USP); Surgicel (TN)	0	0	0	0	0	0	0	0	0	0	0	0	0	0	0	0	0	0	0	0	0	0	0	0	0	0	0	0	0	0	1	0	0	0	0	0
D03497 - Cilengitide (USAN/INN)	0	0	0	0	0	0	0	0	0	0	0	0	0	0	0	0	0	1	0	0	0	0	0	0	0	0	0	0	0	0	0	0	0	0	0	0
D03517 - Cipemastat (USAN/INN); Trocade (TN)	0	0	0	0	0	0	0	0	0	0	0	0	0	0	0	0	0	0	0	0	0	0	0	1	0	0	0	1	0	0	0	0	0	0	0	0
D03692 - Desirudin (USAN/INN); Iprivask (TN)	0	1	0	0	0	0	0	0	0	0	0	0	0	0	0	0	0	0	0	0	0	0	0	0	0	0	0	0	0	0	0	0	0	0	0	0
D03722 - Efegatran sulfate (USAN)	0	1	0	0	0	0	0	0	0	0	0	0	0	0	0	0	0	0	0	0	0	0	0	0	0	0	0	0	0	0	0	0	0	0	0	0
D03728 - Napsagatran (USAN)	0	1	0	0	0	0	0	0	0	0	0	0	0	0	0	0	0	0	0	0	0	0	0	0	0	0	0	0	0	0	0	0	0	0	0	0
D03766 - Diaplasinin (USAN)	0	0	0	0	0	0	0	0	0	0	0	0	0	0	1	0	0	0	0	0	0	0	0	0	0	0	0	0	0	0	0	0	0	0	0	0
D03793 - Ilomastat (USAN/INN)	0	0	0	0	0	0	0	0	0	0	0	0	0	0	0	0	0	0	0	1	0	0	0	1	0	0	0	0	0	0	0	0	0	0	0	0
D03795 - Marimastat (USAN/INN)	0	0	0	0	0	0	0	0	0	0	0	0	0	0	0	0	0	0	0	1	0	0	0	1	0	0	0	0	0	0	0	0	0	0	0	0
D03797 - Prinomastat (USAN/INN)	0	0	0	0	0	0	0	0	0	0	0	0	0	0	0	0	0	0	0	1	0	0	0	1	0	0	0	1	0	0	0	0	0	0	0	0
D03800 - Rebimastat (USAN/INN)	0	0	0	0	0	0	0	0	0	0	0	0	0	0	0	0	0	0	0	1	0	0	0	1	0	0	0	0	0	0	0	0	0	0	0	0
D03802 - Tanomastat (USAN/INN)	0	0	0	0	0	0	0	0	0	0	0	0	0	0	0	0	0	0	0	1	0	0	0	1	0	0	0	0	0	0	0	0	0	0	0	0
D03940 - Eculizumab (USAN/INN); Eculizumab (genetical recombination) (JAN); Soliris (TN)	0	0	0	0	0	0	0	0	1	0	0	0	0	0	0	0	0	0	0	0	0	0	0	0	0	0	0	0	0	0	0	0	0	0	0	0
D04029 - Razaxaban hydrochloride (USAN)	0	0	0	1	0	0	0	0	0	0	0	0	0	0	0	0	0	0	0	0	0	0	0	0	0	0	0	0	0	0	0	0	0	0	0	0
D04183 - Fidexaban (USAN/INN)	0	0	0	1	0	0	0	0	0	0	0	0	0	0	0	0	0	0	0	0	0	0	0	0	0	0	0	0	0	0	0	0	0	0	0	0
D05183 - Ulinastatin (JP18); Miraclid (TN)	0	0	0	0	0	0	0	0	0	0	0	0	0	0	0	0	0	0	1	0	0	0	0	0	0	0	0	0	0	0	0	0	0	0	0	0
D05917 - Stamulumab (USAN)	1	0	0	0	0	0	0	0	0	0	0	0	0	0	0	0	0	0	0	0	0	0	0	0	0	0	0	0	0	0	0	0	0	0	0	0
D06142 - Tifacogin (USAN/INN)	0	0	0	1	0	0	0	0	0	0	0	0	0	0	0	0	0	0	0	0	0	0	0	0	0	0	0	0	0	0	0	0	0	0	0	0

D06160 - Tiplasinin (USAN)	0	0	0	0	0	0	0	0	0	0	0	0	0	0	1	0	0	0	0	0	0	0	0	0	0	0	0	0	0	0	0	0	0	0	0	0
D06283 - Varespladib sodium (USAN)	0	0	0	0	0	0	0	0	0	0	0	0	0	0	0	0	0	0	0	0	0	0	0	0	0	0	0	0	0	0	0	0	1	0	0	0
D06319 - Volociximab (USAN)	0	0	0	0	0	0	0	0	0	0	0	0	0	0	0	0	1	0	0	0	1	0	0	0	0	0	0	0	0	0	0	0	0	0	0	0
D06410 - Thrombomodulin alfa (USAN/INN); Thrombomodulin alfa (genetical recombination) (JAN)	0	1	0	0	0	0	0	0	0	0	0	0	0	0	0	0	0	0	0	0	0	0	0	0	0	0	0	0	0	0	0	0	0	0	0	0
D06456 - Cellulose; oxidized regenerated (USP); Surgicel absorbable hemostat (TN)	0	0	0	0	0	0	0	0	0	0	0	0	0	0	0	0	0	0	0	0	0	0	0	0	0	0	0	0	0	0	1	0	0	0	0	0
D06563 - Belagenpumatucel-L (USAN); Lucanix (TN)	0	0	0	0	0	0	0	0	0	0	0	0	0	0	0	0	0	0	0	0	0	0	0	0	0	0	0	0	1	0	0	0	0	0	0	0
D06634 - Relacatib (USAN/INN)	0	0	0	0	0	0	0	0	0	0	0	0	0	0	0	0	0	0	0	0	0	0	0	0	0	0	1	0	0	0	0	0	0	0	0	0
D06880 - Lepirudin (INN); Refludan (TN)	0	1	0	0	0	0	0	0	0	0	0	0	0	0	0	0	0	0	0	0	0	0	0	0	0	0	0	0	0	0	0	0	0	0	0	0
D07082 - Dabigatran etexilate mesylate (USAN); Dabigatran etexilate methanesulfonate (JAN); Pradaxa (TN)	0	1	0	0	0	0	0	0	0	0	0	0	0	0	0	0	0	0	0	0	0	0	0	0	0	0	0	0	0	0	0	0	0	0	0	0
D07086 - Rivaroxaban (JAN/USAN/INN); Xarelto (TN)	0	0	0	1	0	0	0	0	0	0	0	0	0	0	0	0	0	0	0	0	0	0	0	0	0	0	0	0	0	0	0	0	0	0	0	0
D07143 - Melagatran (INN)	0	1	0	0	0	0	0	0	0	0	0	0	0	0	0	0	0	0	0	0	0	0	0	0	0	0	0	0	0	0	0	0	0	0	0	0
D07144 - Dabigatran etexilate (USAN/INN); Pradaxa (TN)	0	1	0	0	0	0	0	0	0	0	0	0	0	0	0	0	0	0	0	0	0	0	0	0	0	0	0	0	0	0	0	0	0	0	0	0
D07568 - Aminomethylbenzoic acid; p-Aminomethylbenzoic acid; Gumbix (TN)	0	0	0	0	1	0	0	0	0	0	0	0	0	0	0	0	0	0	0	0	0	0	0	0	0	0	0	0	0	0	0	0	0	0	0	0
D08656 - Aleplasinin (USAN/INN)	0	0	0	0	0	0	0	0	0	0	0	0	0	0	1	0	0	0	0	0	0	0	0	0	0	0	0	0	0	0	0	0	0	0	0	0
D08858 - Antithrombin alfa (INN/USAN); Atryn (TN)	0	0	0	1	0	0	0	0	0	0	0	0	0	0	0	0	0	0	0	0	0	0	0	0	0	0	0	0	0	0	0	0	0	0	0	0
D08859 - Apratastat (USAN/INN)	0	0	0	0	0	0	0	0	0	0	0	0	0	0	0	0	0	0	0	0	0	0	0	0	0	0	0	1	0	0	0	0	0	0	0	0
D08873 - Betrixaban (USAN); Bevyxxa (TN)	0	0	0	1	0	0	0	0	0	0	0	0	0	0	0	0	0	0	0	0	0	0	0	0	0	0	0	0	0	0	0	0	0	0	0	0
D08913 - Eribaxaban (USAN)	0	0	0	1	0	0	0	0	0	0	0	0	0	0	0	0	0	0	0	0	0	0	0	0	0	0	0	0	0	0	0	0	0	0	0	0
D08946 - Mipomersen sodium (USAN); Kynamro (TN)	0	0	0	0	0	0	0	0	0	0	0	0	0	1	0	0	0	0	0	0	0	0	0	0	0	0	0	0	0	0	0	0	0	0	0	0
D08955 - Odanacatib (JAN/USAN)	0	0	0	0	0	0	0	0	0	0	0	0	0	0	0	0	0	0	0	0	0	0	0	0	0	0	1	0	0	0	0	0	0	0	0	0
D09342 - Etaracizumab (USAN/INN); Abegrin (TN)	0	0	0	0	0	0	0	0	0	0	0	0	0	0	0	0	0	1	0	0	0	0	0	0	0	0	0	0	0	0	0	0	0	0	0	0
D09403 - Custirsen sodium (USAN)	0	0	0	0	0	0	0	0	0	0	0	0	0	0	0	0	0	0	0	0	0	0	1	0	0	0	0	0	0	0	0	0	0	0	0	0
D09546 - Edoxaban tosilate hydrate (JAN); Edoxaban tosylate monohydrate; Lixiana (TN); Savaysa (TN)	0	0	0	1	0	0	0	0	0	0	0	0	0	0	0	0	0	0	0	0	0	0	0	0	0	0	0	0	0	0	0	0	0	0	0	0
D09620 - Fresolimumab (USAN/INN)	0	0	0	0	0	0	0	0	0	0	0	0	0	0	0	0	0	0	0	0	0	1	0	0	0	0	0	0	1	0	0	0	0	0	0	0
D09631 - Intetumumab (USAN/INN)	0	0	0	0	0	0	0	0	0	0	0	0	0	0	0	0	0	1	0	0	0	0	0	0	0	0	0	0	0	0	0	0	0	0	0	0
D09673 - Tafamidis (JAN/USAN/INN); Vyndamax (TN); Vynmac (TN)	0	0	0	0	0	0	0	0	0	0	0	0	1	0	0	0	0	0	0	0	0	0	0	0	0	0	0	0	0	0	0	0	0	0	0	0
D09674 - Tafamidis meglumine (JAN/USAN); Vyndaqel (TN)	0	0	0	0	0	0	0	0	0	0	0	0	1	0	0	0	0	0	0	0	0	0	0	0	0	0	0	0	0	0	0	0	0	0	0	0
D09707 - Dabigatran (USAN/INN)	0	1	0	0	0	0	0	0	0	0	0	0	0	0	0	0	0	0	0	0	0	0	0	0	0	0	0	0	0	0	0	0	0	0	0	0
D09710 - Edoxaban (USAN/INN)	0	0	0	1	0	0	0	0	0	0	0	0	0	0	0	0	0	0	0	0	0	0	0	0	0	0	0	0	0	0	0	0	0	0	0	0
D09799 - Carotegrast methyl (JAN); Carogra (TN)	0	0	0	0	0	0	0	0	0	0	0	0	0	0	0	0	1	0	0	0	0	0	0	0	0	0	0	0	0	0	0	0	0	0	0	0
D09817 - Darexaban maleate (JAN); YM150	0	0	0	1	0	0	0	0	0	0	0	0	0	0	0	0	0	0	0	0	0	0	0	0	0	0	0	0	0	0	0	0	0	0	0	0
D09903 - Fluciclatide F 18 (USAN/INN)	0	0	0	0	0	0	0	0	0	0	0	0	0	0	0	0	0	1	0	0	0	0	0	0	0	0	0	0	0	0	0	0	0	0	0	0
D09923 - Letaxaban (USAN/INN)	0	0	0	1	0	0	0	0	0	0	0	0	0	0	0	0	0	0	0	0	0	0	0	0	0	0	0	0	0	0	0	0	0	0	0	0
D09929 - Maraciclatide (USAN/INN)	0	0	0	0	0	0	0	0	0	0	0	0	0	0	0	0	0	1	0	0	0	0	0	0	0	0	0	0	0	0	0	0	0	0	0	0
D10023 - Pexelizumab (USAN/INN)	0	0	0	0	0	0	0	0	1	0	0	0	0	0	0	0	0	0	0	0	0	0	0	0	0	0	0	0	0	0	0	0	0	0	0	0
D10028 - Valategrast hydrochloride (USAN)	0	0	0	0	0	0	0	0	0	0	0	0	0	0	0	0	1	0	0	0	0	0	0	0	0	0	0	0	0	0	0	0	0	0	0	0
D10150 - Pegnivacogin sodium (USAN)	0	0	1	0	0	0	0	0	0	0	0	0	0	0	0	0	0	0	0	0	0	0	0	0	0	0	0	0	0	0	0	0	0	0	0	0

D10203 - Trabedersen (INN/BAN)	0	0	0	0	0	0	0	0	0	0	0	0	0	0	0	0	0	0	0	0	0	0	0	0	0	0	0	0	1	0	0	0	0	0	0	0
D10347 - Ramatercept (USAN); Ace-031	1	0	0	0	0	0	0	0	0	0	0	0	0	0	0	0	0	0	0	0	0	0	0	0	0	0	0	0	0	0	0	0	0	0	0	0
D10471 - Idraparinux sodium (USAN)	0	0	0	1	0	0	0	0	0	0	0	0	0	0	0	0	0	0	0	0	0	0	0	0	0	0	0	0	0	0	0	0	0	0	0	0
D10484 - Pegnivacogin (USAN)	0	0	1	0	0	0	0	0	0	0	0	0	0	0	0	0	0	0	0	0	0	0	0	0	0	0	0	0	0	0	0	0	0	0	0	0
D10595 - Asfotase alfa (USAN/INN)	0	0	0	0	0	0	0	0	0	0	0	0	0	0	0	1	0	0	0	0	0	0	0	0	0	0	0	0	0	0	0	0	0	0	0	0
D10794 - Patisiran sodium (JAN); Onpattro (TN)	0	0	0	0	0	0	0	0	0	0	0	0	1	0	0	0	0	0	0	0	0	0	0	0	0	0	0	0	0	0	0	0	0	0	0	0
D10821 - Emicizumab (USAN/INN)	0	0	1	1	0	0	0	0	0	0	0	0	0	0	0	0	0	0	0	0	0	0	0	0	0	0	0	0	0	0	0	0	0	0	0	0
D10913 - Burosumab (USAN/INN)	0	0	0	0	0	0	0	0	0	0	0	0	0	0	0	0	0	0	0	0	0	0	0	0	0	0	0	0	0	0	0	0	0	0	1	0
D10940 - Inotersen (USAN/INN)	0	0	0	0	0	0	0	0	0	0	0	0	1	0	0	0	0	0	0	0	0	0	0	0	0	0	0	0	0	0	0	0	0	0	0	0
D10941 - Inotersen sodium (USAN); Tegsedi (TN)	0	0	0	0	0	0	0	0	0	0	0	0	1	0	0	0	0	0	0	0	0	0	0	0	0	0	0	0	0	0	0	0	0	0	0	0
D10969 - Pamrevlumab (USAN/INN)	0	0	0	0	0	0	0	0	0	0	0	0	0	0	0	0	0	0	0	0	0	0	0	0	0	1	0	0	0	0	0	0	0	0	0	0
D11029 - Andexanet alfa (USAN/INN)	0	0	0	1	0	0	0	0	0	0	0	0	0	0	0	0	0	0	0	0	0	0	0	0	0	0	0	0	0	0	0	0	0	0	0	0
D11032 - Plitidepsin (INN)	0	0	0	0	0	0	0	0	0	0	0	0	0	0	0	0	0	0	0	0	0	0	0	0	0	0	0	0	0	1	0	0	0	0	0	0
D11047 - Domagrozumab (USAN/INN)	1	0	0	0	0	0	0	0	0	0	0	0	0	0	0	0	0	0	0	0	0	0	0	0	0	0	0	0	0	0	0	0	0	0	0	0
D11054 - Ravulizumab (USAN)	0	0	0	0	0	0	0	0	1	0	0	0	0	0	0	0	0	0	0	0	0	0	0	0	0	0	0	0	0	0	0	0	0	0	0	0
D11116 - Patisiran (USAN/INN)	0	0	0	0	0	0	0	0	0	0	0	0	1	0	0	0	0	0	0	0	0	0	0	0	0	0	0	0	0	0	0	0	0	0	0	0
D11235 - Landogrozumab (USAN/INN)	1	0	0	0	0	0	0	0	0	0	0	0	0	0	0	0	0	0	0	0	0	0	0	0	0	0	0	0	0	0	0	0	0	0	0	0
D11242 - Trevogrumab (USAN/INN)	1	0	0	0	0	0	0	0	0	0	0	0	0	0	0	0	0	0	0	0	0	0	0	0	0	0	0	0	0	0	0	0	0	0	0	0
D11262 - Andecaliximab (USAN/INN)	0	0	0	0	0	0	0	0	0	0	0	0	0	0	0	0	0	0	0	0	0	0	0	1	0	0	0	0	0	0	0	0	0	0	0	0
D11265 - Taldefgrobep alfa (USAN); Talditercept alfa (USAN)	1	0	0	0	0	0	0	0	0	0	0	0	0	0	0	0	0	0	0	0	0	0	0	0	0	0	0	0	0	0	0	0	0	0	0	0
D11280 - Bivalirudin trifluoroacetate (USAN); Angiomax (TN)	0	1	0	0	0	0	0	0	0	0	0	0	0	0	0	0	0	0	0	0	0	0	0	0	0	0	0	0	0	0	0	0	0	0	0	0
D11331 - Olendalizumab (USAN); Lendalizumab (USAN)	0	0	0	0	0	0	0	0	1	0	0	0	0	0	0	0	0	0	0	0	0	0	0	0	0	0	0	0	0	0	0	0	0	0	0	0
D11369 - Rosmantuzumab (USAN)	0	0	0	0	0	0	0	0	0	0	0	0	0	0	0	0	0	0	0	0	0	0	0	0	0	0	0	0	0	0	0	0	0	1	0	0
D11464 - Miridesap (USAN/INN)	0	0	0	0	0	0	0	0	0	0	1	0	0	0	0	0	0	0	0	0	0	0	0	0	0	0	0	0	0	0	0	0	0	0	0	0
D11471 - Dezamizumab (USAN/INN)	0	0	0	0	0	0	0	0	0	0	1	0	0	0	0	0	0	0	0	0	0	0	0	0	0	0	0	0	0	0	0	0	0	0	0	0
D11473 - Nomacopan (USAN)	0	0	0	0	0	0	0	0	1	0	0	0	0	0	0	0	0	0	0	0	0	0	0	0	0	0	0	0	0	0	0	0	0	0	0	0
D11477 - Pozelimab (USAN)	0	0	0	0	0	0	0	0	1	0	0	0	0	0	0	0	0	0	0	0	0	0	0	0	0	0	0	0	0	0	0	0	0	0	0	0
D11568 - Edoxaban tosylate (USAN)	0	0	0	1	0	0	0	0	0	0	0	0	0	0	0	0	0	0	0	0	0	0	0	0	0	0	0	0	0	0	0	0	0	0	0	0
D11613 - Pegcetacoplan (JAN/USAN/INN); Empaveli (TN)	0	0	0	0	0	0	0	1	0	0	0	0	0	0	0	0	0	0	0	0	0	0	0	0	0	0	0	0	0	0	0	0	0	0	0	0
D11696 - Crovalimab (USAN/INN)	0	0	0	0	0	0	0	0	1	0	0	0	0	0	0	0	0	0	0	0	0	0	0	0	0	0	0	0	0	0	0	0	0	0	0	0
D11748 - Avacincaptad pegol sodium (USAN)	0	0	0	0	0	0	0	0	1	0	0	0	0	0	0	0	0	0	0	0	0	0	0	0	0	0	0	0	0	0	0	0	0	0	0	0
D11770 - Vonapanitase (USAN/INN)	0	0	0	0	0	0	0	0	0	0	0	0	0	0	0	0	0	0	0	0	0	0	0	0	0	0	0	0	0	0	0	0	0	0	0	1
D11797 - Apitegromab (USAN)	1	0	0	0	0	0	0	0	0	0	0	0	0	0	0	0	0	0	0	0	0	0	0	0	0	0	0	0	0	0	0	0	0	0	0	0
D11838 - Vilobelimab (USAN)	0	0	0	0	0	0	0	0	1	0	0	0	0	0	0	0	0	0	0	0	0	0	0	0	0	0	0	0	0	0	0	0	0	0	0	0
D11916 - Vutrisiran sodium (JAN); Amvuttra (TN)	0	0	0	0	0	0	0	0	0	0	0	0	1	0	0	0	0	0	0	0	0	0	0	0	0	0	0	0	0	0	0	0	0	0	0	0
D11917 - Vutrisiran (USAN/INN)	0	0	0	0	0	0	0	0	0	0	0	0	1	0	0	0	0	0	0	0	0	0	0	0	0	0	0	0	0	0	0	0	0	0	0	0
D11965 - alpha 1-Antitrypsin; Human alpha 1-Proteinase inibitor; freeze-dried concentrated; Lynspad (TN)	0	0	0	0	0	0	1	0	0	0	0	0	0	0	0	0	0	0	0	0	0	0	0	0	0	0	0	0	0	0	0	0	0	0	0	0

D11972 - Acoramidis (USAN)	0	0	0	0	0	0	0	0	0	0	0	0	1	0	0	0	0	0	0	0	0	0	0	0	0	0	0	0	0	0	0	0	0	0	0	0
D11973 - Acoramidis hydrochloride (JAN/USAN)	0	0	0	0	0	0	0	0	0	0	0	0	1	0	0	0	0	0	0	0	0	0	0	0	0	0	0	0	0	0	0	0	0	0	0	0
D12003 - Simufilam (USAN)	0	0	0	0	0	0	0	0	0	0	0	0	0	0	0	0	0	0	0	0	0	0	0	0	1	0	0	0	0	0	0	0	0	0	0	0
D12004 - Simufilam hydrochloride (USAN)	0	0	0	0	0	0	0	0	0	0	0	0	0	0	0	0	0	0	0	0	0	0	0	0	1	0	0	0	0	0	0	0	0	0	0	0
D12149 - Plasminogen; Glu-plasminogen; Plasminogen; human-tvmh; Ryplazim (TN)	0	0	0	0	1	0	0	0	0	0	0	0	0	0	0	0	0	0	0	0	0	0	0	0	0	0	0	0	0	0	0	0	0	0	0	0
D12208 - Gefurulimab (USAN)	0	0	0	0	0	0	0	0	1	0	0	0	0	0	0	0	0	0	0	0	0	0	0	0	0	0	0	0	0	0	0	0	0	0	0	0
D12251 - Iptacopan (USAN/INN)	0	0	0	0	0	1	0	0	0	0	0	0	0	0	0	0	0	0	0	0	0	0	0	0	0	0	0	0	0	0	0	0	0	0	0	0
D12252 - Iptacopan hydrochloride (USAN); Iptacopan hydrochloride monohydrate	0	0	0	0	0	1	0	0	0	0	0	0	0	0	0	0	0	0	0	0	0	0	0	0	0	0	0	0	0	0	0	0	0	0	0	0
D12351 - Tinlarebant (USAN)	0	0	0	0	0	0	0	0	0	0	0	1	0	0	0	0	0	0	0	0	0	0	0	0	0	0	0	0	0	0	0	0	0	0	0	0
D12356 - Zilucoplan sodium (JAN)	0	0	0	0	0	0	0	0	1	0	0	0	0	0	0	0	0	0	0	0	0	0	0	0	0	0	0	0	0	0	0	0	0	0	0	0
D12357 - Zilucoplan (USAN/INN)	0	0	0	0	0	0	0	0	1	0	0	0	0	0	0	0	0	0	0	0	0	0	0	0	0	0	0	0	0	0	0	0	0	0	0	0
D12379 - Galegenimab (USAN)	0	0	0	0	0	0	0	0	0	0	0	0	0	0	0	0	0	0	0	0	0	0	0	0	0	0	0	0	0	0	0	1	0	0	0	0
D12402 - Zinpentraxin alfa (USAN)	0	0	0	0	0	0	0	0	0	0	1	0	0	0	0	0	0	0	0	0	0	0	0	0	0	0	0	0	0	0	0	0	0	0	0	0

REFERENCES

1. Kessels, M. Y. *et al.* Proteomics analysis of the zebrafish skeletal extracellular matrix. *PLoS ONE* **9**, e90568 (2014).
2. Zhang, R., Li, Y. & Xing, X. Comparative antler proteome of sika deer from different developmental stages. *Sci. Rep.* **11**, 10484 (2021).
3. Yao, B. *et al.* Comparative transcriptome analysis of the main beam and brow tine of sika deer antler provides insights into the molecular control of rapid antler growth. *Cell. Mol. Biol. Lett.* **25**, 42 (2020).
4. Frasheri, I. *et al.* Full-length amelogenin influences the differentiation of human dental pulp stem cells. *Stem Cell Res. Ther.* **7**, 10 (2016).
5. Yu, M. *et al.* BMP4 mutations in tooth agenesis and low bone mass. *Arch. Oral Biol.* **103**, 40–46 (2019).
6. Shi, G.-X. *et al.* Evidence of the Role of R-Spondin 1 and Its Receptor Lgr4 in the Transmission of Mechanical Stimuli to Biological Signals for Bone Formation. *Int. J. Mol. Sci.* **18**, (2017).
7. UniProt Consortium. UniProt: the universal protein knowledgebase in 2021. *Nucleic Acids Res.* **49**, D480–D489 (2021).
8. Murphy, G. & Lee, M. H. What are the roles of metalloproteinases in cartilage and bone damage? *Ann. Rheum. Dis.* **64 Suppl 4**, iv44-7 (2005).
9. Borsy, A. *et al.* Identifying novel genes involved in both deer physiological and human pathological osteoporosis. *Mol. Genet. Genomics* **281**, 301–313 (2009).
10. Wang, H. *et al.* R-Spondin 1 promotes vibration-induced bone formation in mouse models of osteoporosis. *J. Mol. Med.* **91**, 1421–1429 (2013).
11. Wang, Y. *et al.* Follistatin-like protein 1: a serum biochemical marker reflecting the severity of joint damage in patients with osteoarthritis. *Arthritis Res. Ther.* **13**, R193 (2011).
12. Jin, Q.-Y. *et al.* Follistatin-like 1 suppresses osteoblast differentiation of bone marrow mesenchymal cells during inflammation. *Arch. Oral Biol.* **135**, 105345 (2022).
13. Chen, P. *et al.* Annexin A1 is a potential biomarker of bone metastasis in small cell lung cancer. *Oncol. Lett.* **21**, 141 (2021).
14. Xu, X. *et al.* Transforming growth factor-β in stem cells and tissue homeostasis. *Bone Res.* **6**, 2 (2018).
15. Du, G. *et al.* TGF-Beta Induced Key Genes of Osteogenic and Adipogenic Differentiation in Human Mesenchymal Stem Cells and MiRNA-mRNA Regulatory Networks. *Front. Genet.* **12**, 759596 (2021).
16. Tiaden, A. N. *et al.* Human serine protease HTRA1 positively regulates osteogenesis of human bone marrow-derived mesenchymal stem cells and mineralization of differentiating bone-forming cells through the modulation of extracellular matrix protein. *Stem Cells* **30**, 2271–2282 (2012).
17. Jiang, W. *et al.* Connective tissue growth factor promotes chemotaxis of preosteoblasts through integrin α5 and Ras during tensile force-induced intramembranous osteogenesis. *Sci. Rep.* **11**, 2368 (2021).
18. Lin, X., Patil, S., Gao, Y.-G. & Qian, A. The bone extracellular matrix in bone formation and regeneration. *Front. Pharmacol.* **11**, 757 (2020).
19. Rosset, E. M. & Bradshaw, A. D. SPARC/osteonectin in mineralized tissue. *Matrix Biol.* **52–54**, 78–87 (2016).
20. Ozaki, Y. *et al.* Neuron-derived neurotrophic factor protects against dexamethasone-induced skeletal muscle atrophy. *Biochem. Biophys. Res. Commun.* **593**, 5–12 (2022).
21. Hopkins, D. R., Keles, S. & Greenspan, D. S. The bone morphogenetic protein 1/Tolloid-like metalloproteinases. *Matrix Biol.* **26**, 508–523 (2007).
22. Zhang, Y., Chen, B., Li, D., Zhou, X. & Chen, Z. LncRNA NEAT1/miR-29b-3p/BMP1 axis promotes osteogenic differentiation in human bone marrow-derived mesenchymal stem cells. *Pathol. Res. Pract.* **215**, 525–531 (2019).
23. Su, Z. *et al.* Overexpression of Bone Morphogenetic Protein-1 Promotes Osteogenesis of Bone Marrow Mesenchymal Stem Cells In Vitro. *Med. Sci. Monit.* **26**, e920122 (2020).
